# Therapist-supported online remote behavioural intervention for tics in children and adolescents in England (ORBIT): a multicentre, parallel group, single-blind, randomised controlled trial

**DOI:** 10.1016/S2215-0366(21)00235-2

**Published:** 2021-10

**Authors:** Chris Hollis, Charlotte L Hall, Rebecca Jones, Louise Marston, Marie Le Novere, Rachael Hunter, Beverley J Brown, Charlotte Sanderson, Per Andrén, Sophie D Bennett, Liam R Chamberlain, E Bethan Davies, Amber Evans, Natalia Kouzoupi, Caitlin McKenzie, Isobel Heyman, Kareem Khan, Joseph Kilgariff, Cristine Glazebrook, David Mataix-Cols, Tara Murphy, Eva Serlachius, Elizabeth Murray

**Affiliations:** a NIHR MindTech MedTech Co-operative, University of Nottingham, Nottingham, UK; b National Institute for Health Research Nottingham Biomedical Research Centre, University of Nottingham, Nottingham, UK; c Mental Health and Clinical Neurosciences, School of Medicine, University of Nottingham, Nottingham, UK; d Department of Child and Adolescent Psychiatry, Nottinghamshire Healthcare NHS Foundation Trust Queen's Medical Centre, Nottingham, UK; e Division of Psychiatry, University College London, London, UK; f Research Department of Primary Care and Population Health, University College London, London, UK; g Priment Clinical Trials Unit, University College London, London, UK; h Great Ormond Street Institute of Child Health, Great Ormond Street Hospital for Children NHS Trust, London, UK; i Psychological and Mental Health Services, Great Ormond Street Hospital for Children NHS Trust, London, UK; j Centre for Psychiatry Research, Department of Clinical Neuroscience, Karolinska Institutet and Stockholm Health Care Services, Region Stockholm, Stockholm, Sweden

## Abstract

**Background:**

Exposure and Response Prevention (ERP) is a form of behavioural therapy for tics; however, its effectiveness remains uncertain. We aimed to evaluate the effectiveness of internet-delivered, therapist-supported, and parent-assisted ERP for treatment of tics in children and young people with Tourette syndrome or chronic tic disorder.

**Methods:**

This multicentre, parallel group, single-blind, randomised controlled trial was conducted across two study sites in England. Participants were recruited via 16 patient identification centres, two study sites in England (Nottingham and London), or online self-referral. Eligible participants were aged 9–17 years, had Tourette syndrome or chronic tic disorder, had not received behavioural therapy for tics in the past 12 months or were about to start, and had a Yale Global Tic Severity Scale (YGTSS) Total Tic Severity Score (TTSS) of more than 15 or more than 10 if they had only motor or vocal tics. Patients were excluded if they had started or stopped medication for tics within the past 2 months; had current alcohol or substance dependence, psychosis, suicidality, anorexia nervosa, or suspected moderate to severe intellectual disability; or presented an immediate risk to self or others; or the parent or carer was unable to speak, read, or write in English. Eligible patients were randomly assigned (1:1) by masked outcome assessors to receive 10 weeks of online, remotely delivered, therapist-supported ERP or psychoeducation (active control). Outcome assessors, statisticians, health economists, the trial manager, and the chief investigator were masked to group allocation. Patients were not directly informed of their allocation, but this could be established from the content once treatment commenced and the patients were not, therefore, considered masked to treatment. The primary outcome was YGTSS-TTSS 3 months after randomisation, and analysis was done in all randomised patients for whom data were available for each timepoint and outcome. Safety analysis was by intention to treat. Longer term follow-up is ongoing. This trial is registered with ISRCTN (ISRCTN70758207) and ClinicalTrials.gov (NCT03483493).

**Findings:**

Between May 8, 2018, and Sept 30, 2019, we assessed 445 candidates for inclusion in the study. 221 potential participants were excluded (90 did not meet inclusion criteria, 84 declined to participate, and 47 unable to contact family). 224 participants were enrolled and randomly assigned to ERP (n=112) or psychoeducation (n=112). The enrolled patients were mostly male (n=177; 79%) and of White ethnicity (n=195; 87%). 11 patients were lost to follow-up 3 months after randomisation in the ERP group, compared with 12 patients in the psychoeducation group. Mean YGTSS-TTSS at 3 months after randomisation was 23·9 (SD 8·2) in the ERP group and 26·8 (7·3) in the psychoeducation group. The mean total decrease in YGTSS-TTSS at 3 months was 4·5 (16%, SD 1·1) in the ERP group versus 1·6 (6%, 1·0) in the psychoeducation group. The estimated mean difference in YGTSS-TTSS change between the groups adjusted for baseline and site was –2·29 points (95% CI –3·86 to –0·71) in favour of ERP, with an effect size of –0·31 (95% CI –0·52 to –0·10). Two serious adverse events occurred (one collapse and one tic attack), both in the psychoeducation group, neither of which were related to study treatment.

**Interpretation:**

ERP is an effective behavioural therapy for tics. Remotely delivered, online ERP with minimal therapist contact time represents an efficient public mental health approach to improve access to behavioural therapy for tics in children and adolescents.

**Funding:**

National Institute for Health Research and Health and Technology Assessment.

**Research in context**

**Evidence before this study**

We searched 21 databases covering medical and health (Embase, MEDLINE, PREMEDLINE In-Process and Other Non-Indexed Citations and PsycINFO, Cochrane Library, Cochrane Central Register of Controlled Trials, Cochrane Database of Systematic Reviews, Database of Abstracts of Reviews of Effectiveness, and Health Technology Assessment), education (Australian Education Index, British Education Index, and Education Resources in Curriculum), social care (Applied Social Sciences Index and Abstracts [ASSIA], International Bibliography of Social Science, Social Sciences Citation Index, Social Services Abstracts, Sociological Abstracts, and Web of Science), and grey literature (Health Management Information Consortium, PsychBOOKS, and PsychEXTRA) for studies published in any language from database inception to Jan 1, 2013. Additional sources were Allied and Complementary Medicine Database, BIOSIS Citation Index, and Cumulative Index to Nursing and Allied Health Literature (CINAHL). We updated this search in 2014 to include studies published to Oct 1, 2014. The search terms comprised subject headings and text words for tic* and tourette* to identify populations with Tourette syndrome or chronic tic disorder. Searches were limited to systematic reviews and randomised controlled trials describing interventions. We identified two randomised controlled trials of habit reversal training or comprehensive behavioural intervention with a total of 133 participants. In 2020, we updated the search again with the same criteria for studies published to Jan 1, 2020, and found no new trials of behavioural interventions for tics in children and adolescents. As part of a systematic review of digital health interventions in 2017, we searched 11 databases (Allied and Complementary Medicine, Ovid, MEDLINE, PsychINFO, PsychARTICLES, Embase, PubMed, ASSIA, Cochrane Library, CINAHL, and Web of Science), with a smaller keyword search of JMIR Publications database, for digital health interventions for mental health disorders in children and young people published between Jan 1, 2013, and Nov 1, 2015. The search strategy collated terms and keywords to identify children and young people (eg, child, adolescent, or young person), mental health disorders, and digital health interventions (eg, internet interventions, apps, eHealth). Full search strategy terms and language restrictions are available in the 2017 paper. We identified 30 unique randomised controlled trials of digital mental health interventions, with no digital intervention studies identified that focused on treatment of tic disorders. As this was a planned review of digital health interventions, these searches were not updated before the current study. Also in 2017, we did a meta-review of scoping, narrative, systematic, or meta-analytical reviews investigating the effectiveness of digital health interventions for mental health problems in children and adolescents. No effective online behavioural interventions for tics have been reported.

**Added value of this study**

To our knowledge, this is the first randomised controlled trial to report the clinical efficacy, safety, and costs of therapist and parent-supported online Exposure and Response Prevention (ERP) behavioural therapy for tics in children aged 9–17 years. We showed that this 10-week online ERP intervention was highly acceptable, well tolerated, and effective in reducing tics 3 months after randomisation, compared with a similar duration of online psychoeducation. The magnitude of the effect on tic reduction was durable, with a slightly greater effect 3 months after treatment ended (6 months after randomisation). Approximately a quarter of the therapist contact time is required compared with face-to-face behavioural therapy to achieve a similar result.

**Implications of all the available evidence**

Digitally enabled ERP for tics is an efficient public mental health approach to increase the reach of an effective treatment for children and adolescents with tic disorders. Further research is needed to establish the optimum care pathways for sequencing and integration of digital and face-to-face behavioural therapy for tics in children and adolescents.

## Introduction

Tic disorders, such as Tourette syndrome or chronic tic disorder, are common conditions that affect up to 1% of young people.^1^ Tics can lead to significant impairment and isolation^2^ and often co-occur with other conditions. Although there are effective pharmacological treatments for tics, these drugs are often associated with side-effects including weight gain and cognitive dulling.^2^ Behavioural therapies for tics include Habit Reversal Training (HRT), in which patients learn to detect tics and use a competing response (an incompatible action) to control them; Comprehensive Behavioural Intervention for Tics (CBIT), which combines HRT with relaxation, functional analysis, and social support; and Exposure and Response Prevention (ERP), in which patients learn to suppress their tics (response prevention) while tolerating urges to tic (exposure). Unlike HRT and CBIT, no competing response is trained in ERP, potentially making it easier to deliver with minimal therapist input.

Evidence of the effectiveness of behavioural therapy for tics is drawn primarily from two large superiority trials of CBIT from the USA in children and adolescents,^3^ and adults.^4^ ERP has been less well evaluated and its effectiveness in treatment for tics compared with an active control intervention is unknown. One small pilot head-to-head comparison^5^ between ERP and HRT in 43 participants reported a similar reduction in tic symptoms for both treatments but was underpowered to show non-inferiority between the treatments.^5^

Although HRT and CBIT have shown similar effectiveness to pharmacotherapy,^2^ and behavioural therapy is recommended as a first line intervention,^1^, ^2^ it is rarely available. In the UK, a small study found that only one in five children and adolescents with tic disorders has access to behavioural therapy, and less than half of those with access received the recommended number of sessions.^6^ Barriers to access include a shortage of trained therapists and therapy being offered only at specialist treatment centres, meaning patients must often travel long distances. The COVID-19 pandemic has highlighted the urgent need to offer cost-effective interventions that can be delivered remotely and in digital formats.^7^

Across various mental health conditions, meta-analyses show that online internet-delivered cognitive behavioural therapy (CBT) is as efficacious as face-to-face delivery and can result in substantial cost-savings.^8^ Evidence from studies^9^ of internet-delivered CBT with adults suggests that therapist-guided online interventions lead to better outcomes than standalone online CBT. Given the paucity of child therapists in this specialty, a more pragmatic solution might entail a blended approach of digitally enabled therapy in which the core therapeutic content is delivered online in a standardised chapter format, but is supported asynchronously by a non-specialist therapist whose primary role is to promote engagement. A meta-analysis^10^ of CBT for anxiety and depression in children suggested that parents are also a potentially valuable but under-researched resource to support the use of internet-delivered CBT in children.

Researchers in Sweden developed an online platform to provide therapist-supported internet-delivered CBT, called BIP (Barninternetprojektet [Child Internet Project]). The platform has been used to deliver therapy to children with a range of mental health conditions, including anxiety and obsessive-compulsive disorder.^11^, ^12^ Compared with other conditions, internet delivery of therapy for tics has received less interest.^13^ One small pilot study^14^ using the BIP platform compared 10 weeks of therapist-guided, parent-supported HRT against ERP in children and adolescents with tics who were followed up at 3 months and 12 months after end of treatment. The findings indicted that the method of delivery was highly acceptable to families. Although this pilot study was not designed or powered to evaluate efficacy, the results support the feasibility and promise of online ERP for treating tics and justify further investigation to assess clinical effectiveness and cost-effectiveness. In our study, we aimed to evaluate the clinical effectiveness and costs of a therapist-supported, parent-assisted, internet-delivered ERP programme for tics in children and adolescents in England.

## Methods

### Study design

The Online Remote Behavioural Intervention for Tics (ORBIT) trial is a multicentre, parallel group, single-blind, randomised controlled trial, done across two Child and Adolescent Mental Health Services sites in England. Site one (Queen's Medical Centre, Nottingham) was based in a mid-sized city and was a regional centre for tic treatment. Site two (Great Ormond Street Hospital, London) was a large metropolitan national paediatric centre of excellence. The trial had two phases: phase 1 was a per-protocol follow-up for 6 months after patient randomisation and phase 2 is a naturalistic follow-up for 18 months after patient randomisation. We present the findings from phase 1.

Ethical and Health Research Authority approval was received from North West Greater Manchester Research Ethics Committee (18/NW/0079). The published trial protocol is available online^15^ and was approved by an independent trial steering committee and data monitoring committee. Two substantial amendments were made (appendix p 1).

### Participants

Eligible participants were aged 9–17 years with a moderate or severe tic disorder (Tourette syndrome or chronic tic disorder) defined as Yale Global Tic Severity Scale (YGTSS)^16^ Total Tic Severity Score (TTSS) of more than 15, or more than 10 if only motor or vocal tics were present in the past 7 days. All participants were required to have broadband and access to smartphone, desktop computer, or laptop computer and the capacity to provide informed, written consent.

Participants were excluded if they had engaged in structured behavioural intervention for tics (eg, HRT, CBIT, or ERP) within the previous 12 months or were about to start; started or stopped medication for tics within the previous 2 months; had current alcohol or substance dependence, psychosis, suicidality, anorexia nervosa (assessed via the Development and Well Being Assessment [DAWBA]),^17^ or suspected moderate to severe intellectual disability (assessed via Child and Adolescent Intellectual Disability Screening [CAIDS-Q]);^18^ presented an immediate risk to self or others; or the child had no parent or carer who was able to speak, read, or write in English.

Participants were recruited either by referral from one of 16 participating Child and Adolescent Mental Health Services or community paediatric clinics in England and the two study sites, or by self-referral via the Tourettes Action website or the study website. The study outcome assessors completed an initial telephone consultation to establish likely eligibility, and parents or carers completed the online DAWBA. Potential participants who were deemed eligible after this initial screening phase attended a baseline assessment at one of the two study sites, at which assessors did further eligibility assessments, including YGTSS and CAIDS-Q. Written informed consent was obtained from participants or their parents or carers before undertaking the baseline assessment.

Participants younger than 16 years had parent or guardian signed written consent and signed their own written assent. Participants aged 16–17 years had parent or guardian signed written consent and signed their own written consent.

### Randomisation and masking

Participants were randomly assigned (1:1) to receive either 10 weeks of online, remotely delivered, therapist-supported ERP for tics, or online therapist-supported education about tics (referred to as psychoeducation [the active control]). Outcome assessors randomly assigned participants using a secure web-based randomisation system developed by Sealed Envelope and managed by Priment Clinical Trials Unit, following specified standard operating procedures. Randomisation was stratified by study site using block randomisation with varying block sizes. Therapists and an independent assessor who did not do outcome assessments were informed of the allocation via email. The independent assessor verified that each participant was assigned to their allocated intervention, and no instances of incorrect allocation were observed. Outcome assessors, statisticians, health economists, the trial manager, and the chief investigator were masked to group allocation. Participants were not directly informed of their allocation by either the researcher or the therapist, but they might have been able to establish allocation from the content once treatment commenced. Participants were reminded about the importance of masking at each follow-up, and breaks of allocation concealment were reported to the trial manager. All instances would be reviewed by the independent trial steering committee and data monitoring committee.

### Procedures

Treatments were delivered via the secure online BIP platform. Participants and their parent or carers created their log-in details at the baseline assessment and set a treatment start date within 1 week of random assignment. Where possible, participants were briefly introduced to their therapist in person at the baseline assessment. Preliminary measures were done, including demographics, Child and Adolescent Service Use Schedule,^19^ and Children's Global Assessment Scale (CGAS),^20^ exposure to concomitant interventions and CAIDS-Q (to establish eligibility for treatment). We then did the YGTSS assessment to establish presence and severity of tics as per the protocol inclusion criteria. All researchers undertook extensive YGTSS training and supervision sessions. Training and supervision were delivered by a clinician expert in the delivery and evaluation of the primary measures. All researchers had to reach a specified level of expertise before initiation of baseline assessments. The therapists assigned the participant to their allocated treatment and emailed a reminder to log in on their start date.

The treatment content has been described elsewhere.^15^ In summary, information was presented in chapters, which the family (child or adolescent and parent or carer) were requested to work through. The therapist aimed to have 10–20 minutes contact time with the participants (combined contact time with the parent or carer and child or adolescent) each week to check progress, encourage motivation, and answer questions, but did not deliver therapeutic content. Both the ERP intervention and the psychoeducation consisted of ten chapters for the child or adolescent and ten different chapters for parents or carers, designed to be delivered over 10 weeks. The therapist provided support for either ERP or psychoeducation through asynchronous contact (typically delivered via online messages sent through BIP) during these 10 weeks.

The internet-delivered ERP intervention was adapted from published treatment manuals by Verdellen and colleagues.^21^ Participants were requested first to practise controlling their tics for increasingly long periods of time (response prevention), and then to deliberately provoke the premonitory urges while not releasing any tics (exposure and response prevention). All tics were targeted at the same time. Specific triggers to provoke the urge to tic were identified and used by participants, and then used in everyday situations to improve generalisability of the gains. The psychoeducation comparator focused on the history, prevalence, and risk typically associated with tic disorders, and advised healthy habits with no information on tic control. For both interventions, the main treatment information was delivered via ten child-completed chapters; participants were considered treatment completers if child chapters 1–4 were completed. The first four chapters included the active exposure and response prevention components of the intervention and were thus considered the minimum therapeutic dose. The ten parent chapters focused on how best to support the child during their treatment.

Therapists (graduate level education) were not required to have previous experience in treating tic disorders but were trained on the platform and its contents and received regular expert supervision.^22^ As the therapists did not deliver active therapeutic content it was not necessary to account for potential therapist effects in statistical analysis. Participants completed brief online measures at 3 weeks and 5 weeks after randomisation, and completed online and outcome-assessor rated measures at baseline, 3 months, 6 months, 12 months, and 18 months after randomisation. All follow-up outcome-assessor rated measures were completed remotely, via videoconferencing or telephone.

### Outcomes

The primary outcome was tic severity at 3 months after randomisation as measured by YGTSS-TTSS, a semistructured interview that combines separate scales of motor tics (score 0–25) and vocal tics (score 0–25) to provide a total score of 0–50, with higher scores indicating greater severity. YGTSS-TTSS is the gold standard measure of tics and is used widely in clinical practice and research. It is freely available in the public domain and has been translated into many languages. YGTSS-TTSS was completed by a masked outcome-assessor. All outcome-assessors completed mandatory structured training on YGTSS before starting, and agreement with an expert rater was assessed every 6 months (appendix p 2).

Secondary outcomes were reduction in tic-related impairment assessed through the YGTSS impairment scale (score 0–50); global assessment of symptom improvement measured via the Clinical Global Impressions—Improvement scale (CGI-I);^23^ global functioning assessed via CGAS; and service use using a modified version of the Child and Adolescent Service Use Schedule to include specific specialist tic disorder services and medications. These outcomes were measured at baseline, 3 months, and 6 months after randomisation, through interviews done by the masked outcome assessors with the parent or carer and child or young person.

Parents or carers reported secondary outcomes online, including measures of general behavioural and emotional difficulties (Strengths and Difficulties Questionnaire),^24^ generic health-related quality of life (proxy-rated child-health-utility-9D [CHU9D]),^25^ and adverse events or side-effects (modified version of the Hill and Taylor^26^ side-effects scale). A parent assessment of tics measured via the Parent Tic Questionnaire^27^ was completed at these times and at 5 weeks after randomisation.

Additional outcomes completed online by the child or adolescent included generic quality of life by CHU9D and a disease-specific measure of quality of life by Child and Adolescent Gilles de la Tourette Syndrome Quality of Life Scale (C&A-GTS-QOL).^28^ Two additional measures (Mood and Feelings Questionnaire^29^ and Spence Childhood Anxiety Scale)^30^ were completed by the child or adolescent at 5 weeks after randomisation and at baseline, 3 months, and 6 months after randomisation. For the purpose of this study, a measure of treatment credibility was developed and completed online by parent or carer and child or adolescent at 3 weeks.^15^ Premonitory urges were recorded at baseline using the Premonitory Urge for Tics Scale.^31^

Adverse events and side-effects were formally sought and recorded at each follow-up by the side-effects scale and Mood and Feelings Questionnaire. Participants were also encouraged to report adverse effects to their therapist or outcome assessor (appendix pp 4–6).

### Statistical analysis

Based on findings of other trials,^1^ we calculated the sample size to detect a clinically important average difference of 0·5 SDs between ERP and psychoeducation with 90% power at p<0·05 (two-sided). When allowing for 20% dropout, this required a total sample size of 220 participants.

Statistical analyses were done using Stata (version 16) in line with a predefined statistical analysis plan approved by the trial steering committee. Analysis for the primary outcome was done on a modifed intention-to-treat basis, in which participants were analysed according to their allocated group using all available data for a given outcome and timepoint. In line with the statistical analysis plan, 95% CIs are reported rather than p values. Secondary outcomes and duration of response were analysed based on number of patients in the intention-to-treat population who supplied data. Safety analyses were done by intention to treat.

Baseline demographic characteristics of participants and their clinical and mental health outcomes at baseline, 3 months after randomisation, and 6 months after randomisation were summarised by randomised group using mean (SD) for continuous data or count (%) for categorical data. The primary outcome was estimated using a linear regression model with YGTSS-TTSS at 3 months as the outcome and study group as the main explanatory variable, adjusting for YGTSS-TTSS at baseline and site.

Similar linear regression models were fitted to estimate the effect of the intervention on secondary outcomes at 5 weeks, 3 months, and 6 months after random assignment. The statistical model for CGI-I did not adjust for baseline since this is a measure of change. Using CGI-I to indicate response to treatment, the scale was dichotomised to define response as improved or much improved versus non-response as minimally improved, stayed the same, worse, or very much worse. Two unplanned subgroup analyses explored whether the effect of the intervention on the primary outcome was modified by diagnosis of either anxiety or ADHD. The statistical models were the same as for the main analysis of the primary outcome, with the addition of a fixed effect of the comorbidity (anxiety or ADHD) and an interaction between the comorbidity and study groups. All statistical analyses were by complete case.

A text message notification was sent to the therapist every time a participant or parent logged in so they could monitor progress and provide support if needed. A variable cost was calculated at £0·17 for each SMS notification. A separate full economic evaluation will be done at 18 months of follow-up (phase 2) as the follow-up duration of 6 months is insufficient for calculating an incremental cost per quality-adjusted life year (QALY) gained. Here we explore the cost of delivering the ERP and psychoeducation interventions, examine relevant health-care resource use, and evaluate the suitability of CHU9D for calculating QALYs in an 18-month analysis. Further details on the method and findings can be found in the appendix (pp 8–23). The trial was registered with ISRCTN (ISRCTN70758207) and ClinicalTrials.gov (NCT03483493).

### Role of the funding source

The study was funded by the UK National Institute for Health Research Health Technology Assessment (Ref 16/17/02). The funders had no role in the study design, data collection, data analysis, data interpretation, writing of the report, or the decision to submit the paper for publication.

## Results

**Figure 1:**
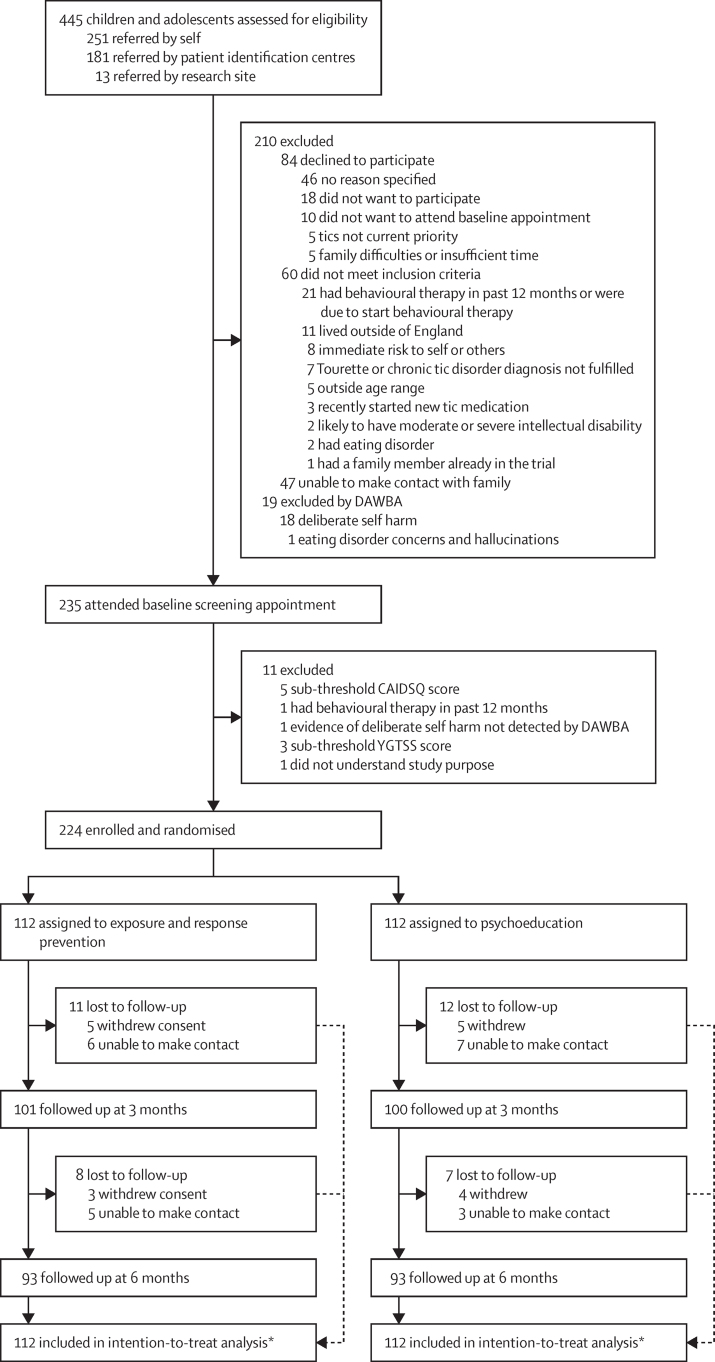
Trial profile DAWBA=Development and well-being assessment. CAIDS-Q=Child and Adolescent Intellectual Disability Screening Questionnaire. YGTSS=Yale Global Tic Severity Scale. *Analysis was done in all randomised patients for whom data were available for each timepoint and outcome. Safety analysis was by intention to treat.

Between May 8, 2018, and Sept 30, 2019, we assessed 445 potential participants for inclusion in the study. 210 were excluded following initial telephone screening (n=191) or DAWBA results (n=19), and 235 attended a baseline assessment and gave informed consent. 11 candidates were excluded after the further screening measures, and 224 participants were enrolled and randomly assigned to receive ERP intervention (n=112) or psychoeducation (n=112; figure 1). 204 patients received the minimum intervention (completing at least the first four chapters) and were considered treatment completers (99 in the ERP group and 105 in the psychoeducation group). In the ERP group, 11 patients were lost to follow-up 3 months after randomisation, and a further eight patients were lost to follow-up 6 months after randomisation. In the psychoeducation group, 12 patients were lost to follow-up 3 months after randomisation, and a further seven were lost to follow-up 6 months after randomisation. 186 patients were followed up 6 months after randomisation (93 in the ERP group and 93 in the psychoeducation group). Although participants were reminded about the importance of masking at each follow-up, four instances of allocation concealment breaking were reported to the trial manager. In all instances the child disclosed information about their treatment to the outcome assessor at the end of the follow-up assessment. In these cases, subsequent follow-up assessments were done by an alternative, masked assessor. All instances were reviewed by the independent trial steering committee and data monitoring committee for monitoring. The last participant completed the 6-month follow-up on April 30, 2020, at which point phase 1 of the ORBIT trial was completed.

Baseline characteristics are shown in table 1. Participants had a mean age of 12 years, were predominately male (177 [79%] of 224) and defined their ethnicity as White (195 [87%]). Only 30 (13%) participants were receiving medication for tics. Premonitory urges score at baseline was similar across the groups (mean Premonitory Urge for Tics Scale score 22, SD 7 for the ERP group and 21, SD 6 for the psychoeducation group), equating to medium intensity for premonitory urges. Baseline scores on the primary and secondary outcome measures were similar across the trial groups (table 2). Primary outcome data were available for 99 (88%) of 112 participants in the intervention group and 105 (94%) of 112 participants in the psychoeducation group. The only predictor of missingness was site, which was included as a covariate in the statistical model.

**Table 1:** Baseline characteristics of participants

		**Psychoeducation (n=112)**	**Exposure and response prevention (n=112)**
	Age at random assignment, years	12·4 (2·1)	12·2 (2·0)
Sex			
	Male	87 (78%)	90 (80%)
	Female	25 (22%)	22 (20%)
Ethnicity			
	White	99 (88%)	96 (86%)
	Asian	3 (3%)	7 (6%)
	Black	0 (0%)	1 (1%)
	Mixed	7 (6%)	3 (3%)
	Other	1 (1%)	0 (0%)
	Not given	2 (2%)	5 (4%)
Main caregiver in trial			
	Mother	101 (90%)	93 (83%)
	Father	10 (9%)	16 (14%)
	Grandmother	1 (1%)	1 (1%)
	Other	0 (0%)	2 (2%)
Mother's highest educational level			
	No qualifications	1 (1%)	3 (3%)
	Mandatory secondary education (eg, GCSEs)	17 (15%)	16 (14%)
	Further education (eg, A-levels, BTEC, NVQ)	32 (29%)	33 (29%)
	Higher education (eg, BA, BSc)	46 (41%)	46 (41%)
	Postgraduate education (eg, MA, MSc, PhD)	16 (14%)	14 (13%)
Father's highest educational level			
	No qualifications	5 (4%)	2 (2%)
	Mandatory secondary education (eg, GCSEs)	29 (26%)	29 (26%)
	Further education (eg, A-levels, BTEC, NVQ)	33 (29%)	35 (31%)
	Higher education (eg, BA, BSc)	34 (30%)	32 (29%)
	Postgraduate education (eg, MA, MSc, PhD)	11 (9%)	14 (13%)
Mother's occupational status			
	Not in work or unemployed	22 (20%)	19 (20%)
	Manual or semimanual occupation	26 (23%)	24 (21%)
	Professional occupation	57 (51%)	65 (58%)
	Other	7 (6%)	4 (4%)
Father's occupational status			
	Not in work or unemployed	4 (4%)	2 (2%)
	Manual or semimanual occupation	30 (27%)	33 (29%)
	Professional occupation	67 (60%)	65 (58%)
	Other	10 (9%)	12 (11%)
Tic types			
	Both motor and vocal tics	106 (95%)	103 (92%)
	Motor tics only	6 (5%)	9 (8%)
	Vocal tics only	0 (0%)	0 (0%)
Comorbidities*			
	Anxiety disorder†	27 (24%)	34 (30%)
	ADHD	25 (22%)	26 (23%)
	Oppositional defiant disorder	23 (21%) of 111	26 (24%) of 110
	Autism spectrum disorder	4 (4%)	9 (8%) of 111
	Obsessive compulsive disorder	3 (3%)	8 (7%)
	Major depression	6 (5%)	2 (2%)
	Conduct disorder	2 (2%) of 111	3 (3%) of 110
Taking any medication for tics‡		16 (13%)	14 (13%)
Centre			
	Nottingham	57 (51%)	57 (51%)
	London	55 (49%)	55 (49%)

Data mean (SD) or n (%).

* Comorbidities are based on 50% or greater probability of having a DSM-IV or DSM 5 diagnosis as assessed by the Development and Wellbeing Assessment. Denominators vary because insufficient information was supplied for some participants to make either a positive or negative diagnosis.

† Anxiety disorders included were separation anxiety, specific phobias, social phobia, panic disorder, agoraphobia, and post-traumatic stress disorder. Diagnoses are not mutually exclusive and so percentages are not expected to total 100%.

‡ Clonidine, risperidone, aripiprazole, haloperidol, guanfacine, or topiramate.

**Table 2:** Primary and secondary outcome scores at baseline and 3 and 6 months after randomisation

		**Psychoeducation (n=112)**	**Exposure and response prevention (n=112)**	**Estimated difference (95% CI)**	**Standardised effect size**
**Baseline**					
Primary outcome					
	YGTSS-TTSS	28·4 (7.·1)	28·4 (7·7)	..	..
Secondary outcomes					
	YGTSS-Impairment	22·9 (9·9)	23·8 (10·3)	..	..
	Parent Tic Questionnaire	53·1 (26·1)	54·7 (29·9)	..	..
	Children's Global Assessment Scale	72·1 (11·8)	70·7 (13·7)	..	..
	Strengths and Difficulties Questionnaire	16·3 (6·2)	18·0 (6·5)	..	..
	Mood and Feelings Questionnaire	15·9 (11·5)	16·3 (11·3)	..	..
	Spence Child Anxiety Scale*	30·5 (17·9)	32·9 (20·2)	..	..
	C&A-GTS-QOL†	35·0 (17·2)	36·6 (16·4)	..	..
**3 months**					
Primary outcome					
	YGTSS-TTSS	26·8 (7·3)	23·9 (8·2)	−2·29 (−3·86 to −0·71)	−0·31 (−0·52 to −0·10)
Secondary outcomes					
	Patients analysed for secondary outcomes at 3 months (n)	101	100	..	..
	YGTSS-Impairment	19·1 (10·9)	16·7 (10·4)	−2·24 (−4·82 to 0·33)	..
	Parent Tic Questionnaire	45·7 (25·5)	34·7 (26·4)	−9·44 (−15·37 to −3·51)	..
	Clinical Global Impression-Improvement	3·37 (1·11)	2·96 (1·1)	−0·41 (−0·71 to −0·11)	..
	Children's Global Assessment Scale	75·2 (12·6)	75·9 (12·6)	0·96 (−1·48 to 3·41)	..
	Strengths and Difficulties Questionnaire	14·2 (6·3)	14·7 (6·1)	−0·38 (−1·62 to 0·85)	..
	Mood and Feelings Questionnaire	12·6 (11·1)	10·7 (11·1)	−1·36 (−3·75 to 1·02)	..
	Spence Child Anxiety Scale	28·2 (18·3)	27·2 (19·0)	−2·80 (−6·52 to 0·93)	..
	C&A-GTS-QOL†	31·8 (17·7)	25·7 (18·0)	−4·81 (−8·79 to −0·83)	..
**6 months**					
Primary outcome					
	YGTSS-TTSS	25·0 (7·6)	21·5 (8·8)	−2·64 (−4·56 to −0·73)	−0·36 (−0·62 to −0·10)
Secondary outcomes					
	Patients analysed for secondary outcomes at 6 months (n)	93	93	..	..
	YGTSS-Impairment	17·0 (10·5)	14·7 (10·7)	−1·95 (−4·68 to 0·78)	..
	Parent Tic Questionnaire	40·6 (24·3)	31·1 (21·6)	−8·60 (−14·43 to −2·77)	..
	Clinical Global Impression Scale–Improvement	3·1 (1·1)	2·8 (1·3)	−0·31 (−0·66 to 0·03)	..
	Children's Global Assessment Scale	76·8 (12·3)	77·5 (14·7)	0·60 (−2·24 to 3·44)	..
	Strengths and Difficulties Questionnaire	13·3 (6·1)	15·3 (6·2)	0·57 (−0·93 to 2·07)	..
	Mood and Feelings Questionnaire	11·4 (11·2)	11·4 (12·1)	−0·61 (−3·85 to 2·64)	..
	Spence Child Anxiety Scale	25·9 (18·7)	25·7 (19·6)	−5·10 (−9·70 to −0·50)	..
	C&A-GTS-QOL†	28·9 (18·3)	27·4 (16·5)	−2·91 (−7·60 to 1·78)	..

Data are mean (SD) unless otherwise specified and are calculated for all available data. YGTSS=Yale Global Tic Severity Scale. TTSS=Total Tic Severity Score. C&A-GTS-QOL=Child and Adolescent Gilles de la Tourette Syndrome–Quality of Life Scale. Statistical models were adjusted for the baseline measure of the outcome in question (except the Clinical Global Impressions Scale-Improvement) and site. For the standardised effect size, YGTSS-TTSS was standardised by the pooled mean and SD at baseline. 3 months after randomisation, there were 12 missing observations (11%) for the primary outcome in the Exposure and Response Prevention group compared with 11 (10%) in the psychoeducation group. The quantity of missing data for secondary outcomes was similar between groups.

* One value missing at baseline.

† Higher scores for C&A-GTS-QOL indicate worse quality of life.

Mean YGTSS-TTSS at 3 months after randomisation was 23·9 (SD 8·2) in the ERP group compared with 26·8 (7·3) in the psychoeducation group. The mean total decrease in YGTSS-TTSS at 3 months was 4·5 (16%, SD 1·1) in the ERP group versus 1·6 (6%, 1·0) in the psychoeducation group, and at 6 months was 6·9 (24%, 1·2) in the ERP group versus 3·4 (12%, 1·0) in the psychoeducation group. The estimated mean difference in YGTSS-TTSS change between the groups at 3 months, adjusted for baseline and site, was –2·29 points (95% CI –3·86 to –0·71) in favour of ERP, with an effect size of –0·31 (95% CI –0·52 to –0·10; table 2).

**Figure 2:**
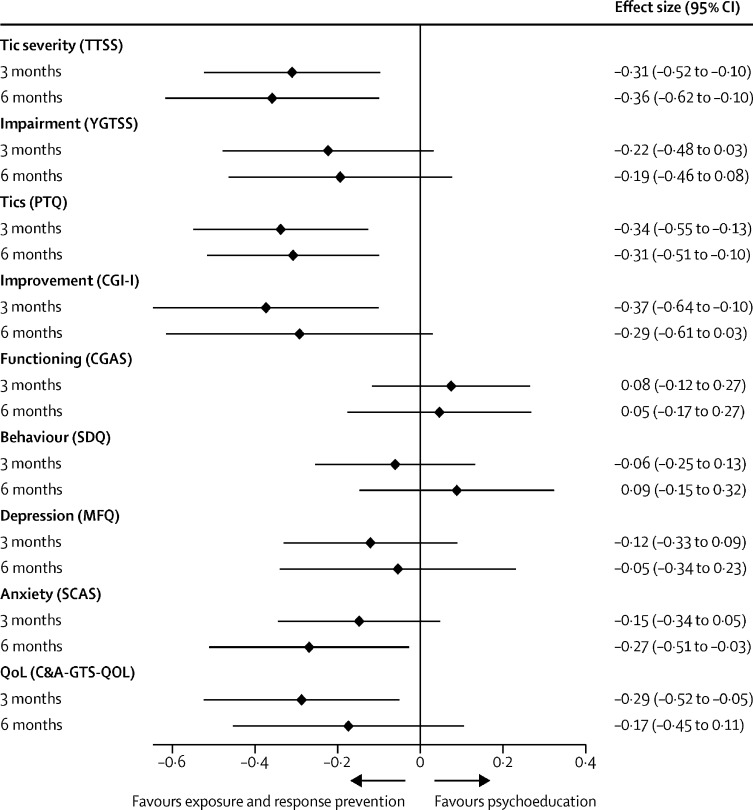
Standardised effect sizes for primary and secondary outcomes TTSS=Total Tic Severity Score. YGTSS=Yale Global Tic Severity Scale. PTQ=Parent Tic Questionnaire. CGIS-I=Clinical Global Impressions-Improvement. CGAS=Children's Global Assessment Scale. SDQ=Strengths and Difficulties Questionnaire. MFQ=Mood and Feelings Questionnaire. SCAS=Spence Child Anxiety Scale. QoL=Quality of Life. C&A-GTS-QOL=Child and Adolescent Gilles de la Tourette Syndrome-Quality of Life Scale.

This adjusted effect on tics increased slightly from 3 months to 6 months (estimated difference in YGTSS-TTSS –2·64, 95% CI –4·56 to –0·73; effect size –0·36, –0·62 to –0·10). Standardised effect sizes for primary and secondary outcomes are shown in figure 2.

The secondary outcome of parent-reported tic symptoms (by Parent Tic Questionnaire) supported the primary outcome finding at 3 months (estimated difference between treatment groups –9·44, 95% CI –15·37 to –3·51) and 6 months (−8·60, –14·43 to –2·77). There was no statistically significant difference in tic-related impairment as measured by the YGTSS impairment scale at either timepoint (table 2).

Other secondary outcomes including parent-reported general emotional and behavioural functioning, young person-reported low mood, and outcome assessor-reported overall functioning were not significantly different between the two groups at 3 or 6 months. Although there was no difference in young person-reported anxiety at 3 months, there was a difference in favour of the ERP group at 6 months (estimated difference in Spence Childhood Anxiety Scale –5·10, 95% CI –9·70 to –0·50). Young person-reported tic-specific quality of life (C&A-GTS-QOL) and the outcome assessor-completed perception of global improvement (CGI-I) showed better results in the ERP group than psychoeducation at 3 months (estimated difference in C&A GTS-QOL –4·81, 95% CI –8·79 to –0·83; estimated difference in CGI-I –0·41; 95% CI –0·71 to –0·11) but showed no difference at 6 months.

There were no differences in score on the Mood and Feelings Questionnaire or Parent Tic Questionnaire at 5 weeks after randomisation (appendix p 26). An unplanned post-hoc analysis found no evidence that the ERP therapy had a different effect in participants with or without a comorbid anxiety disorder or with or without comorbid ADHD (appendix p 26). An unplanned analysis showed a significantly greater positive treatment response with ERP at 3 months (patients with CGI-I rating 1 or 2 [very much or much improved] 36 [36%] of 101 patients) than with psychoeducation (20 [20%] of 100 patients, odds ratio [OR] 2·22; 95% CI 1·17–4·20, table 3). This superior treatment response was sustained at 6 months for ERP (44 [47%] of 93 participants) compared with psychoeducation (27 [29%] of 93 participants, OR 2·20; 95% CI 1·20–4·04). There were more responders in the ERP group at both timepoints (table 3).

**Table 3:** Response to treatment at 3 months and 6 months after randomisation

	**Psychoeducation**	**Exposure and response prevention**	**Odds ratio (95% CI)**
**Baseline**			
Patients randomly assigned	112	112	..
**3 months**			
Patients with available follow-up data	100	101	..
CGI-I score indicating much or very much improved (responded to treatment)	20 (20%)	36 (36%)	2·22 (1·17–4·20)
**6 months**			
Patients with available follow-up data	93	93	..
CGI-I score indicating much or very much improved (responded to treatment)	27 (29%)	44 (47%)	2·20 (1·20–4·04)
**Change in response between 3 and 6 months**			
Patients with available follow-up data	93	90	..
No response to treatment at either time	56 (60%)	37 (41%)	..
Response at both times	9 (10%)	23 (26%)	..
New responder at 6 months	18 (19%)	20 (22%)	..
Relapsed responder at 6 months	10 (11%)	10 (11%)	..

Data are n or n (%). Statistical models were adjusted for site. Percentages are calculated by how many participants were still being followed up at each timepoint and for whom follow-up data were available (ie, completion of questionnaires or outcome measures). CGI-I=Clinical Global Impressions–Improvement.

Two serious adverse events were recorded during the trial, affecting two participants in the psychoeducation group (one male participant collapsed and was admitted to hospital due to a functional movement disorder and one female participant attended accident and emergency due to a tic attack). Both participants were discharged from hospital with no further action. The parents or carers of the two participants stated that they did not feel the events had been brought on by trial participation, and participants had not recently engaged with the internet-delivered content when the events occurred. Both serious adverse events were reviewed by the independent trial steering committee and data monitoring committee and deemed unrelated to trial participation. Slightly fewer adverse events were reported in the ERP group (n=359) than in the psychoeducation group (n=431), and fewer participants in the ERP group had one or more adverse events (88 [79%] of 112 participants) than in the psychoeducation group (94 [84%] of 112 participants). The most commonly occurring adverse events were low mood, increase in tics, and anger or irritability (appendix p 6).

Overall, engagement with the intervention was high in both groups, with minimum treatment completion (at least four chapters) in 99 (88%) of 112 participants in the ERP group and 105 (94%) of 112 participants in the psychoeducation group. The number of log-ins were similar across both groups, with only slightly more log-ins for the participants in the ERP group (mean difference 7·07, 95% CI 2·27 to 11·88; p=0·02, appendix p 7). Perception of treatment suitability and credibility was also high across both groups (appendix p 7). Patients in the ERP group had a mean of 14·27 minutes more therapist time per participant than did patients in the psychoeducation group (95% CI –1·80 to 30·36, p=0·082), but therapist time required to effectively support ERP was low (approximately 2·5 h contact time per participant, 15 minutes per week combined child or young person and parent or carer contact time).

The fixed yearly cost of delivering the intervention was £103·64 per participant (yearly cost of BIP platform £8494 and total cost of supervision and training £14 719·78). As the interventions were delivered on the same platform there was no difference in fixed costs.

There was a small but significant difference between the two groups in the variable costs of the platform resulting from more platform logins and slightly more therapist contact time in the ERP intervention group (appendix pp 13–15; table 4). There were no significant differences in wider health-care costs (appendix p 16). As a combination of the fixed and variable costs and including wider health-care costs, delivering the ERP intervention cost £159 (95% CI 53–370) more per participant than did psychoeducation.

**Table 4:** Variable costs between the psychoeducation and exposure and response prevention groups across 6 months

		Psychoeducation (n=111)*	Exposure and response prevention (n=111)*	**Difference**
Cost of therapist contact time				
	Young person	£16 (9)	£18 (9)	..
	Parent or carer 1	£22 (10)	£25 (13)	..
	Parent or carer 2	£0·09 (0·56)	£0·20 (2)	..
	Total	£38 (17)	£43 (20)	£4·99 (0·01–9·96), p=0·049
Login costs				
	Young person	£3 (1)	£3 (2)	..
	Parent or carer 1	£3 (2)	£3 (2)	..
	Parent or carer 2	£0·01 (0·10)	£0·04 (0·33)	..
	Total	£6 (3)	£7 (4)	£1·25 (0·46–2·04), p=0·00
Total variable costs		£44 (18)	£50 (22)	£6·27 (0·88–11·67), p=0·02

Data are mean (SD) or estimated difference (95% CI).

* One patient in each group had no therapist time.

## Discussion

Online delivery of therapist-supported ERP is an effective treatment of tics in children and adolescents. To our knowledge, ORBIT is the first adequately powered, randomised, controlled trial assessing ERP for tics. ORBIT also represents the largest trial of any behavioural treatment for tics and the first trial to examine the effectiveness of an online internet-delivered behavioural intervention for tics in children and adolescents compared with an active control condition. The trial recruited ahead of time and target, reflecting a substantial unmet treatment need in the population. A particular strength of the design was the inclusion of an active comparator arm controlling for non-specific effects of therapist contact, homework assignments, and online access. The uptake of both the ERP and psychoeducation was excellent, as was retention to the primary outcome at 3 months (90%) and 6 months after randomisation (>80%). Acceptability and safety of the intervention were high. Analysis of our primary outcome (tic severity at 3 months after random assignment) indicated a significant effect in favour of therapist-supported ERP compared with supported psychoeducation. Importantly, the therapeutic effect was durable and even increased slightly at 6 months. Compared with the psychoeducation comparator, participants were twice as likely to show a positive treatment response with the ERP intervention.

The participants in this trial had moderate to severe baseline tic severity, which was approximately 0·5 SD higher than reported in previous face-to-face behavioural treatment trials.^2^, ^5^ The trial design minimised the clinical comorbidity exclusions, resulting in a sample broadly representative of real-world clinical practice, and included participants with autism spectrum disorder, a group usually excluded in similar behavioural intervention trials. In the behavioural intervention group, just under a third had a coexisting anxiety disorder and just under a quarter had ADHD. The reduction in tics associated with the behavioural intervention was similar in those with and without co-existing anxiety or ADHD diagnoses.

In our previous systematic review^2^ of tic treatments in children and adolescents, we identified two superiority trials^2^, ^4^ of face-to-face behavioural therapy (HRT or CBIT) for tics that reported a medium effect in improving tics in favour of behavioural therapy compared with waitlist or supportive psychotherapy (pooled effect size 0·64, 95% CI 0·29–0·99). The magnitude of effect of this online ERP in the current trial is about half that reported from previous superiority trials of face-to-face HRT or CBIT for tics.^2^ However, it is difficult to make direct comparisons of therapeutic efficacy with previous trials of face-to-face behavioural therapy given that this trial had higher baseline tic severity, fewer comorbidity exclusions, a lower proportion of participants receiving tic medication, longer follow-up, and a potent active comparator. In practice, direct comparison of efficacy might also be misleading with respect to implementation because the purpose is not to replace face-to-face therapy, but to allow this scarce resource to be better targeted to those who need it most, and to offer an effective digitally enabled intervention to a much larger population of children and adolescents who are unable to access any behavioural treatment for tics.

A major difference between online delivery and face-to-face behavioural therapy for tics is the reduced amount of therapist time, the required skill level of the therapist, and cost. The total therapist contact time in this trial was around 2·5 h compared with 9–10 h in comparable evidence-based face-to-face behavioural therapy for tics. Given the shortage of highly trained therapists with expertise in tic disorders and scarce access to behavioural therapy, online delivery of ERP for tics has the potential to greatly expand the reach of effective behavioural interventions. With more efficient use of therapist time it should be possible to treat four people for every one person treated with face-to-face therapy. Moreover, the lower level of experience required in therapists to support online behavioural therapy should expand the potential pool of therapists and thereby further extend availability. A strength of the online delivery model is that fidelity of therapeutic content is built into the intervention, making transfer to real-world effectiveness much less susceptible to therapeutic drift and the skill level of individual therapists than in traditional face-to-face therapy.

The study has several limitations. First, it is the first adequately powered trial of therapist-supported, online ERP, and replications are required. Second, it is not possible in this trial to separate the effects of digital online delivery and ERP. In future, clinical and cost-effectiveness comparisons of digital online versus face-to-face ERP or CBIT will be needed. Third, some people do not have sufficient access to the internet and smartphones, which could have limited the reach of this internet-delivered ERP intervention. Although this does not appear to be an issue in the UK, wherein 90% of households have access to the internet and 98% of young people own a smartphone,^32^ it could be an important consideration when generalising these findings to other countries or populations with less access. Fourth, a large proportion of the sample was White, which could limit the generalisability of the findings with regards to ethnicity. Fifth, the amount of tic medication use and co-morbid obsessive compulsive disorder diagnoses were lower than in comparable studies done in the USA, which might limit generalisability to these populations. Finally, although tic severity in ORBIT is higher than in comparable studies, the findings might not be generalisable to those young people with tics outside the severity range of this study population.

Implementation research will be required to establish how best to integrate online behavioural therapy for tics within treatment pathways. For example, digital or online delivery might work best as a first-line behavioural intervention, with non-responders or poor responders being stepped-up to more intensive face-to-face therapy. Another model to evaluate would be the blending of online and face-to-face therapy for more complex cases, thereby reducing the overall number of face-to-face sessions required.

Evidence from this trial suggests that online therapist-supported ERP is an effective behavioural therapy for reduction in tic symptoms which has the potential to greatly increase the availability of effective behavioural treatment for children and adolescents with tic disorders.

## Data sharing

De-identified individual participant data that underlie the results reported in this article (text, tables, figures, data dictionary, and appendices) will be available. The full study protocol, statistical analysis plan, informed consent forms, and patient information sheets will be available beginning 3 months and ending 5 years after article publication. Researchers who provide a methodologically sound proposal should direct these to priment@ucl.ac.uk, and data requestors will need to sign a data access agreement.

## Declaration of interests

CH was Principal Investigator on a grant from the National Institute of Health Research (NIHR) Health Technology Assessment programme to do an Evidence Synthesis on the treatments for tics and Tourette syndrome in children and young people (HTA Project 10/142/01). DM-C reports personal fees from Elsevier and personal fees from UpToDate outside the submitted work. All other authors received funding from NIHR to support their salaries during the conduct of the study.
