# Correction to *Lancet Psychiatry* 2021; 8: 871–82

**DOI:** 10.1016/S2215-0366(21)00464-8

**Published:** 2022-01

*Hollis C, Hall CL, Jones R, et al. Therapist-supported online remote behavioural intervention for tics in children and adolescents in England (ORBIT): a multicentre, parallel group, single-blind, randomised controlled trial.* Lancet Psychiatry 2021; 8: *871–82*—In the Results section of this Article, the number of participants for whom primary outcome data were available should have been 101 participants in the intervention group and 100 participants in the psychoeducation group. This correction has been made to the online version as of Nov 22, 2021.

