# Correction to *Lancet Psychiatry* 2021; 8: 871–82

**DOI:** 10.1016/S2215-0366(21)00477-6

**Published:** 2022-06

*Hollis C, Hall CL, Jones R, et al. Therapist-supported online remote behavioural intervention for tics in children and adolescents in England (ORBIT): a multicentre, parallel group, single-blind, randomised controlled trial.* Lancet Psychiatry *2021;* 8: *871–82*—In table 2 of this Article, the number of participants for whom outcome data were available should have been for primary outcome analysis, and at 3 months these data should have read 101 participants in the intervention group and 100 participants in the psychoeducation group. This correction has been made to the online version as of Dec 3, 2021.

