# Supplementary material for: Therapist-supported online remote behavioural intervention for tics in children and adolescents in England (ORBIT): a multicentre, parallel group, single-blind, randomised controlled trial
Source: Lancet Psychiatry. 2021 Oct;8(10):871–82. doi: 10.1016/S2215-0366(21)00235-2 (PMC8460453; doi:10.1016/S2215-0366(21)00235-2)
Supplement: Supplementary appendix [file mmc1.pdf]

THE LANCET
Psychiatry

Supplementary appendix

This appendix formed part of the original submission and has been peer reviewed. We post it as supplied by the authors.

Supplement to: Hollis C, Hall CL, Jones R, et al. Therapist-supported online remote behavioural intervention for tics in children and adolescents in England (ORBIT): a multicentre, parallel group, single-blind, randomised controlled trial. *Lancet Psychiatry* 2021; published online Sept 1. http://dx.doi.org/10.1016/S2215-0366(21)00235-2.

Substantial amendments

REC reference: 18/NW/0079

Amendment number 1: Substantial amendment number 1.0

Date: 19th April 2018 Approved: 15th May 2018

Description of amendment:

Since the initial application the protocol has since been registered with clinicaltrials.gov and the ISRTCN. These registration details have been added to the protocol (IRAS reference Section A5 1: Research reference numbers). In our initial protocol we stated that the therapist-supported intervention would be delivered over a 10-week period (IRAS form: Section A13-Summary of methodology and design and Section A19-Details of the intervention).

Since the submission of our approved protocol, our project management team met and discussed concerns of access to the therapist during periods of bank holidays/set leave (such as Christmas) which may influence the amount of therapist contact during the 10-week intervention. We consulted with our PPI group who mentioned that even if a therapist was available, families may not be able to have dedicated time to participate in the therapy over the Christmas period, which may also apply to periods such as exam time, Easter, or illness.

Our Project Management Group (including members from our PPI panel) propose to amend the protocol to allow the therapist guided treatment to be given over a 12 week period, as long as the therapist only offers support for a maximum of 10 weeks during the 12 week period. This may be needed if the participant is unable to engage with the ORBIT treatment for a period of time for reasons such as, holidays, exam periods, illness or bereavement.

If any circumstance occurs meaning the child is unable to login and access the ORBIT treatment for 5 days or more, therapist support and access to the treatment should be paused for that week, until the child is able to fully engage in the treatment again. Treatment and therapist support can be paused for a maximum of two weeks. Therapists will consult with the trial manager and their clinical supervisor for these cases.

Amendment number 2: Substantial amendment number 1.0

Date: 28th June 2018 Approved: 16th July 2018

Description of amendment (not related to current paper):

In the protocol, we mentioned we will be conducting four semi-structured interviews with clinicians, therapists, children, and parents as part of the process evaluation. The interviews explore their experiences of being a part of the trial and experiences of the intervention.

However, at the time of original submission of the protocol we did not submit the interview schedules. Having undergone PPI review, the four interview schedules are now ready to be submitted. The interviews are already mentioned in the information sheets and in the consent forms. As such, no changes to these documents are required and no additional consent forms are needed.

ORBIT plan for agreement criteria on the YGTSS (primary outcome)

Yale Global Tic Severity Scale

The primary outcome for the ORBIT Trial is the severity of tics as measured by the total tic severity score (TTSS; 0-50) on the Yale Global Tic Severity Scale (YGTSS;[1]). The YGTSS is administered by a blinded outcome-assessor as an investigator-based semi-structured interview focussing on motor and vocal tic frequency, severity and tic related impairment over the previous week. The YGTSS symptom checklist lists 46 tic disorder symptoms, including 12 simple motor tics (e.g., eye blinking), 19 complex motor tics (e.g., facial expressions), seven simple vocal tics (e.g., coughing), and eight complex vocal tics (e.g., words), with four of these items designated on the instrument as "other" symptoms. The YGTSS generates a total tic severity score (0-50) and an impairment score (0-50).

Five index scores are obtained: Total Motor Tic Score, Total Phonic Tic Score, Total Tic Score, Overall Impairment Rating, and Global Severity Score. The Total Motor Tic Score is derived by adding the five items pertaining to motor tics (range: 0–25); the Total Phonic Tic Score is derived by adding the five items pertaining to phonic tics (range: 0–25); the Total Tic Score is derived by adding the Total Motor Tic Score and the Total Phonic Tic Score; and the Overall Impairment Rating is rated on a 50-point scale anchored by 0 (*no impairment*) and 50 (*severe impairment*). A Global Severity Score (range: 0–100) is derived by summing the Total Motor Tic Score, Total Phonic Tic Score, and Overall Impairment Rating. The Total Tic Severity Score (0 - 50) is the primary outcome.

YGTSS Rater training

For ORBIT, rater training of our YGTSS assessors will consist of the following steps:

1) Researchers training on the YGTSS will be supervised by Dr Tara Murphy (TM) the Expert Rater (ER).

2) Training will consist of reading TM's slides on YGTSS which include the background and basic instructions for using the YGTSS.

3) They are also required to read the pivotal Leckman et al. (1988) paper.

4) Assessors are asked to view at least 3 pre-recorded YGTSS assessment sessions. They will be asked to rate these, however this will be solely for training purposes, with discussions afterwards and scores will not be used to calculate reliability or agreement. Assessors are encouraged to make a list of any questions/queries and rationale behind their decision making.

5) They then discuss their scores on this with TM.

6) Assessors then undergo a live test score with TM (i.e. the score a dummy patient) and also shadow a live assessment of YGTSS with an experienced assessor and score the patient. These scores are then discussed with TM/experienced assessor to compare scores and discuss the rationale behind the scoring.

7) The next step will be a testing phase where 3 different YGTSS assessments will be used against an ER to determine the extent of agreement with the ER.

8) In line with the methodology reported by [2] the raters have to be within 15% of the ER for the **Total Motor Tic score**, the **Total Vocal Tic score** and the **Total Tic Score** on the 3 recordings.

9) The 15% will always be rounded up in cases where 15% of a score results in a score which is not a whole integer (i.e. 15% of 25 = 3.75 points, this would be rounded up to 4 points). The 15% can be in either direction of the score.

10) Assessors who do not meet the criteria will be given additional training and asked to score the recordings again until the specified agreement criteria are met.

11) The assessors agreement with the ER will be assessed every 6 months during the trial. Up to a total of 4 new YGTSS assessment videos will be used to check agreement at follow-up (i.e. after initial agreement within the specified range has been established). The videos will be recordings from YGTSS conducted on ORBIT patients at baseline or follow-up assessments. Each of the trial assessors will submit at least one video.

12) If any assessors falls outside this 15% agreement with the ER, they will be required to engage in further training, as and when this is appropriate during the trial.

13) Assessors also participate in monthly conference calls with the ER and trial manager. These calls provided a forum for discussing cases and developing a common approach to conducting assessments across sites. Additional reviewing of videos and discussion with the assessors about scoring will be provided by the ER via email on an ad hoc basis. The ER will keep a record of these supervision meetings.

References

1. Leckman JF, Riddle MA, Hardin MT, et al. The Yale Global Tic Severity Scale: initial testing of a clinician-rated scale of tic severity. *J Am Acad Child Psy* 1989; **28**(4): 566-73.
2. Jeon S, Walkup JT, Woods DW, et al. Detecting a clinically meaningful change in tic severity in Tourette syndrome: a comparison of three methods. *Contemp Clin Trials* 2013; **36**(2): 414-20.

Table 1. Results of training and six-monthly checks assessors scores

	TTS	TTS 15 low	TTS 15 high	Motor total	Motor 15low	Motor 15high	Vocal total	Vocal 15low	Vocal 15high
**Rater Baseline Training Scores**									
Video 1									
ER	26	22˙1	29˙9	15	12˙8	17˙3	11	9˙4	12˙7
A1	29	22˙1	29˙9	15	12˙8	17˙3	**14**	9˙4	12˙7
A2	**30**	22˙1	29˙9	17	12˙8	17˙3	**13**	9. 4	12˙7
A3	29	22˙1	29˙9	16	12˙8	17˙3	**13**	9. 4	12˙7
A4	**30**	22˙1	29˙9	17	12˙8	17˙3	**13**	9. 4	12˙7
A5	26	22˙1	29˙9	13	12˙8	17˙3	**13**	9. 4	12˙7
Video 2									
ER	27	22˙9	31˙1	15	12. 8	17˙3	12	10˙2	13˙8
A1	28	22˙9	31˙1	16	12˙8	17˙3	12	10˙2	13˙8
A2	30	22˙9	31˙1	16	12˙8	17. 3	**14**	10˙2	13˙8
A3	**33**	22˙9	31˙1	**20**	12˙8	17. 3	13	10˙2	13˙8
A4	27	22˙9	31˙1	16	12˙8	17. 3	11	10˙2	13˙8
A5	28	22˙9	31˙1	17	12˙8	17. 3	11	10˙2	13˙8
Video 3									
ER	26	22˙1	29˙9	11	9˙4	12. 7	15	12˙8	17˙3
A1	26	22˙1	29˙9	12	9˙4	12. 7	14	12˙8	17˙3
A2	**39**	22˙1	29˙9	**19**	9˙4	12. 7	**20**	12˙8	17. 3
A3	**35**	22˙1	29˙9	**19**	9˙4	12. 7	16	12˙8	17. 3

	TTS	TTS 15 low	TTS 15 high	Motor total	Motor 15low	Motor 15high	Vocal total	Vocal 15low	Vocal 15high
A4	**32**	22·1	29·9	**17**	9·4	12. 7	15	12·8	17. 3
A5	**36**	22·1	29·9	**19**	9·4	12. 7	17	12·8	17. 3
Video 4									
ER	22	18·7	25·3	15	12·8	17·3	7	6	8
A1	24	18·7	25·3	17	12·8	17·3	7	6	8
A2	Left position on trial – no further YGTSS conducted								
A3	25	18·7	25·3	**18**	12·8	17·3	7	6	8
A4	23	18·7	25·3	15	12·8	17·3	8	6	8
A5	24	18·7	25·3	17	12·8	17·3	7	6	8
**Rater Expert Agreement Check January 2019**									
ER	34	28·9	39 ·1	17	14·5	19·6	17	14·5	19·6
A1	37	28·9	39·1	**21**	14·5	19·6	16	14·5	19·6
ER	10	8·5	11·5	10	8·5	11·5	0	0	0·2
A3	11	8·5	11·5	11	8·5	11·5	0	0	0·2
ER	29	24·7	33·4	13	11·1	15	16	13·6	18·4
A4	29	24·7	33·4	13	11·1	15	16	13·6	18·4
ER	31	26·4	35·7	15	12·8	17·3	16	13·6	18·4
A5	29	26·4	35·7	14	12·8	17·3	15	13·6	18·4
**Rater Expert Agreement Check June 2019**									
ER	35	29·8	40·3	17	14·5	19·6	18	15·3	20·7
A1	33	29·8	40·3	18	14·5	19·6	15	15·3	20·7
ER	30	25·5	34·5	16	13·6	18·4	14	11·9	16·1
A3	29	25·5	34·5	17	13·6	18·4	12	11· 9	16·1
ER	20	17	23	10	8·5	11·5	10	8·5	11·5
A4	23	17	23	**13**	8·5	11·5	10	8·5	11·5
ER	47	39·9	54·1	23	19·6	26·5	24	20·4	27·6
A5	45	39·9	54·1	23	19·6	26·5	22	20·4	27·6
**Rater Expert Agreement Check December 2019 (A3-A4 no longer actively conducting YGTSS)**									
ER	34	28·9	39·1	18	15·3	20·1	16	13·6	18·4
A1	31	28·9	39·1	18	15·3	20·1	**13**	13·6	18·4
ER	22	18·7	35·3	13	11·1	14·9	9	7·7	10·4
A1	**28**	18·7	35·3	**17**	11·1	14·9	**11**	7·7	10·4
ER	33	28·1	37·9	17	14·5	19·6	16	13·6	18·4
A5	33	28·1	37·9	17	14·5	19·6	16	13·6	18·4
ER	25	21·3	28·8	16	13·6	18·4	9	7·7	10·4
A5	25	21·3	28·8	15	13·6	18·4	10	7·7	10·4
**Retraining given to R1 and scores on new videos December 2019**									
ER	34	28·9	39·1	18	15·3	20·7	16	13·6	18·4
A1	31	28·9	39·1	18	15·3	20·7	15	13·6	18·4
ER	22	18·7	25·3	13	11·1	14·9	9	7·7	10·4
A1	25	18·7	25·3	15	11·1	14·9	10	7·7	10·4
**Rater Expert Agreement Check June 2020**									
ER	24	20·4	27·6	14	11· 9	16·1	10	8·5	11·5
A1	25	20·4	27·6	16	11·9	16·1	9	8·5	11·5
ER	12	10·2	13·8	12	10·2	13·8	0	0	0
R1	12	10·2	13·8	12	10·2	13·8	0	0	0
ER	19	16·2	21·9	11	9·4	12·7	8	6·8	9·2
A5	19	16·2	21·9	10	9·4	12·7	9	6·8	9·2
ER	41	34·9	47·2	21	17·9	24·2	20	17	23

	TTS	TTS 15 low	TTS 15 high	Motor total	Motor 15low	Motor 15high	Vocal total	Vocal 15low	Vocal 15high
A5	38	34.9	47.2	18	17.9	24.2	20	17	23

Note. ER = expert rater. R=researcher. TTS = total tic score. 15low = 15% lowest threshold. 15high = 15% highest threshold. Motor total = total motor tic score. Vocal total = total vocal tic score. Scores in bold are outside the threshold

Adverse Events

Adverse events (AEs) and serious adverse events (SAEs) are recorded until the 6-month follow-up and may be reported through various sources in this study:

1) AE's/SAEs may be reported to the therapist or the researcher through communication with the participants.
2) AE's are also recoded through completion of the Mood & Feelings Questionnaire (MFQ), completed at baseline, 5weeks, and 3-6-12-18-months. Any total score >29 at the follow-ups (5weeks, 3months, 6months) or any score greater than 0 on items 16, 17 and 19 (indicators of suicidal ideation) at follow-ups (5weeks, 3months, 6months) is recorded as an AE **if** it is greater than their baseline score. The MFQ is completed by the young person.
3) AE's are also recorded through the side-effects questionnaire completed at baseline, 5weeks, 3 and 6-months. A score on any item that is equal-to-or-greater-than 2 ("about half the time") **and** greater than their baseline score is recorded as an AE. The side-effects questionnaire is completed by the parent/carer.

AEs are recorded by the trial manager and researchers. Seriousness is assessed in the first instance by the trial manager and the researchers who reported the AE, if there are any concerns or doubts with seriousness or relation to the intervention the Chief Investigator (CI) is informed. The CI is informed of all SAEs and is responsibility for making final categorisation.

The protocol defines an SAE as any untoward occurrence that:

- Results in death,
- Is life-threatening,
- Requires hospitalisation or prolongation of existing hospitalisation,
- Results in persistent or significant disability or incapacity, or
- Consists of a congenital anomaly or birth defect
- Is otherwise considered medically significant by the investigator

The following are a list of adverse events listed as 'expected' in the protocol.

- Increased anger/outbursts/disruptive behaviour
- Increased irritability
- Increased depressed mood
- Increased anxiety/stress
- Increased tics
- Increased tiredness/fatigue
- Headaches
- Increased/decreased sleep

Table 1. **Adverse events recorded throughout ORBIT Trial**

	Psychoeducation (control group) (N = 112)	ERP (intervention group) (N = 112)
**Number of events**	**N**	**N**
Serious adverse events	2	0
All adverse events (including serious)	433	359
Expected adverse events (including serious) by type		
Low mood / depressed	78	57
Increased tics	47	36
Increased anger / outbursts / disruptive behaviours	37	21
Increased irritability	41	45
Increased anxiety / stress	21	26
Increased tiredness / fatigue	2	2
Headaches	13	16
Increased / decreased sleep	52	38
Unexpected adverse events (including serious) by type		
Daydreaming	22	14
Increased OCB / OCD type behaviours	1	1
Excited	35	32
Nightmares	11	12
Unsteady	10	1
Poor appetite	13	15
Talks less to / less interested in other children	35	27
Stomach ache	11	16
Restlessness	1	0
Changes in focus	2	0
Unspecified	1	0
Sex differences		
Adverse events - Male	311	289
Adverse events - Females	120	70
Serious adverse events – Male	1	0
Serious adverse events - Female	1	0
**Number of participants**	**N (%)**	**N (%)**
Experiencing a serious adverse event	2 (2%)	0 (0%)
Experiencing any adverse event (including serious)	94 (84%)	88 (79%)

***Notes**: Statistics are n (%) unless otherwise specified. Percentages are given to the nearest whole number. OCB = obsessive compulsive behaviour; OCD = obsessive compulsive disorder.*

Table 2. **Engagement with the intervention and perception of treatment suitability and credibility**

	**Psychoeducation (control) (N = 112)**	**ERP (intervention) (N = 112)**
Young person met therapist – N (%)	86 (77%)	80 (71%)
Treatment suitability and expectation of improvement (credibility) – Median (25th to 75th centiles)		
Young person	6 (5 to 7)	7 (6 to 8)
Supporting parent/caregiver	5 (4 to 6)	6 (5 to 7)
Completion of first four chapters of intervention (adherence) – N (%)		
Young person	105 (94%)	99 (88%)
Supporting parent/caregiver	103 (92%)	95 (85%)
Number of chapters of intervention completed (dose) – Median (25th to 75th centiles)		
Young person	9 (7 to 10)	8 (6 to 10)
Supporting parent/caregiver	10 (8 to 10)	9 (5 to 10)
Number of logins – Median (25th to 75th centiles)		
Young person	13 (10 to 18)	19 (10 to 28)
Parent/ carer	17 (12 to 24)	18 (12 to 27)
**Therapist time on platform in minutes mean (SD)**		
Young person	55·6 (27·1)	59·2 (29·2)
Parent/carer 1	74·2 (34·4)	83·9 (42·8)
Parent/ carer 2	0·4 (2·1)	1·0 (7·2)
**Therapist time on phone in minutes mean (SD)**		
Young person	0·1 (0·3)	0·2 (1·8)
Parent/ carer 1	3·5 (7·5)	3·7 (6·4)
Parent/ carer 2	0·3 (2·0)	0·2 (1·9)
**Total therapist time in minutes mean (SD)**	133·9 (55·1)	148·2 (64·9)
**Platform logins mean (SD)**		
Young person	14·6 (8·6)	19·8 (10·9)
Parent/ carer 1	18·1 (9·0)	20·4 (11·5)
Parent/ carer 2	1·6 (2·6)	6·8 (8·7)

Notes: *Statistics are as specified. IQR = interquartile range. Treatment completers defined as young person completion of first four chapters. ERP = exposure and response prevention.*

Health economic analysis and results

Aim

The aim of the economic evaluation is to calculate the cost of online-delivered, therapist-supported ERP compared with online education from a health and social care cost perspective over 6 months. This will be reported alongside the difference in Total Tic Severity Score (TTSS) on the Yale Global Tic Severity Scale (YGTSS) to calculate the mean incremental cost per point reduction in YGTSS-TTSS.

Secondary aims are to:

- Report descriptive statistics for resource use;
- Calculate the incremental cost per point difference in YGTSS-TTSS from a wider cost perspective.
- Report descriptive statistics for utility tariffs calculated using responses to the CHU-9D over 6 months to inform the design of a decision analytical model of online ERP vs online education at 18 months.

Outputs

- Mean fixed cost per participant of the web-based platform for those in the ERP arm
- Mean cost per participant of variable platform costs and therapist support by trial arm
- Mean total health care cost per participant over 6 months by trial arm
- Mean incremental cost per point decrease in YGTSS-TTSS of Online ERP compared with Online Education and 95% confidence intervals
- Cost-effectiveness plane
- Cost-effectiveness acceptability curve
- Mean utility per participant at baseline, 3 and 6 months by trial arm

Methods

The cost of the platform is split into a fixed cost per participant and a variable cost based on platform use.

<u>Fixed Cost:</u>

A yearly cost of the platform was provided by the Swedish team in SEK. This was translated into GBP using the exchange rate at the time of the analysis (September 2020). This cost is divided by the number of participants using the platform to a conservative estimate of the fixed cost per participant: it is likely that there will be a higher caseload of people using the platform if it were to be rolled out and hence it is likely the cost per patient would be less.

<u>Variable Cost:</u>

Every time a participant logs into the BiP platform they are sent a text message. To account for this an average of the cost of sending a text with different UK network providers has been calculated. This has then been multiplied by the number of log ins to the platform by participants and their parent/carer.

Cost of delivering therapist support

The cost of therapist support is also broken down into a fixed and variable cost.

<u>Fixed Cost:</u>

The fixed cost of therapist support is made up of the cost of training and supervision for the therapists. Most of the therapists were trained in supervision which occurred every week over the course of the intervention for an hour. The hourly cost of therapists and those providing supervision is used to calculate the total cost of supervision and training. This cost is also divided by the number of participants using the platform in order to provide an average fixed cost of therapist support per participant. It is also likely that this is a conservative estimate (the true cost per patient is likely to be less as the number of patients per therapist may be higher).

Variable Cost:

Total therapist time per participant is collected as part of the platform. This is broken down into therapist time for the child, parent 1 and parent 2, and phone time with the therapist for the child, parents and parents and child together. A patient level variable cost of therapist support is calculated for each participant based on therapist time reported and the grade of the therapist they are reported to have interacted with.

Total Health and Social Care and wider societal costs

Health and social care resource use is collected using an adapted version of the Child and Adolescent Service Use Schedule (CA-SUS) developed in previous studies.[1,2,3] Resource use is collected on specialist tic services, contacts with professionals in the community, inpatient contacts, emergency contacts and medication use. The number of participants reporting each type of resource use are reported as a proportion of their trial arm as well as the average number of contacts for those that used the service.

The total cost of health and social care resource use is calculated using unit costs from the most recent Unit costs of Health and Social Care published by the personal Social Services Research unit (PSSRU)[4] and reference costs. Medication costs are calculated using the British National Formulary (BNF). Mean cost per participant in the ERP group versus the psychoeducation (active control) is reported as total mean cost per participant and by type of service at baseline and total at 6 months. The difference in costs at 6 months and 95% confidence intervals are calculated using a bias corrected bootstrapped regression adjusted for baseline and site. Costs are not discounted as the time horizon is less than one year.

Unit costs that were used in the analysis are reported in Table 1. Where contacts occurred at home a ratio of direct to indirect time was applied based on the ratio of GP home visits to clinic visits.[4] Similarly, an average of the ratio from GP clinic contacts to GP phone contacts and nurse clinic contacts to nurse phone contacts was applied to adapt unit costs to phone contacts when the individual cost of phone contacts was not available.

***Table 1.* Unit costs for specialist tic services**

Item	Unit cost (£)	Source
**Tic Disorder clinic**		
Specialist assessment	949	Cost provided by clinicians at GOSH and NUH
Treatment session in clinic	67	Cost provided by clinicians at GOSH and NUH
**CAMHS**		
Multidisciplinary team	119	Cost from PSSRU 2016 adjusted using NHS cost inflation index
**Paediatrician (hospital)**	180	average of consultant and non-consultant led hospital paediatrician from PSSRU 2019
**Paediatrician (community)**	176	Average of consultant and non-consultant led community paediatrician PSSRU 2019
**Child and Adolescent Psychiatrist**	292	Weighted average of child and adolescent psychiatry national reference costs 2019
**Neurologist**	109	Medical Consultant, hospital-based doctors (PSSRU 2019)
**Psychologist**	54	Band 7 community based clinical psychologist (PSSRU 2019)
**Speech and language therapist**	54	Band 7 community-based speech and language therapist (PSSRU 2019)
**Occupational Therapist**	45	Band 6 community-based occupational therapist (PSSRU 2019)
**Rheumatologist**	354	Paediatric rheumatology (national reference costs)
**ENT**	105	Weighted average ENT attendances (national reference costs)
**Geneticist**	368	Geneticist (national reference costs)
**Counsellor**	54	Band 7 community scientific and professional staff (PSSRU 2019)
**School counsellor**	49	School based children’s health services (PSSRU 2019)
**School based speech and language intervention**	91	Average of group based and 1 to 1 session (PSSRU 2019)
**School nurse**	59	School based children’s health services (PSSRU 2019)
**Podiatry**	67	Weighted average podiatry attendances (PSSRU 2019)
**Cardiologist**	179	Weighted average paediatric attendances (national reference costs)
**Psychiatric Nurse**	55	Nurse (mental health clinic) (PSSRU 2019)
**Orthotic services**	86	Weighted average orthotics attendances (national reference costs)
**Orthopaedics**	128	Weighted average paediatric orthopaedics (national reference costs)
**Dentist**	133	Dentist patient contact (PSSRU 2019)
**Endocrinologist**	228	Weighted average paediatric endocrinologist (national reference costs)

Table 2. **Unit costs for contacts with professionals in the community**

**General Practitioner**		
Clinic	39	9-minute GP appointment (PSSRU 2019)
**Nurse (GP practice)**		
Clinic	6	10-minute GP nurse appointment (PSSRU 2019)
**Social worker**	50	Children's services (PSSRU 2019)
**Special education need co-ordinator**	21	Cost per hour based on average salary from National careers service, 37-hour work week and 46.4 working weeks per year.
**Educational psychologist**	54	Band 7 community psychologist
**Parental group**	26	Average of cost per session from different parental groups in PSSRU 2019.
**Play/art/music/drama therapist**	54	Band 7 art therapist in community based scientific and professional staff (PSSRU 2019)
**Physiotherapist**		
Group session	81	NHS costs for children's health services (PSSRU 2019)
One-to-one	100	
**Dietitian**	90	National reference costs
**Osteopath**	40	NHS England website on osteopath
**Orthodontics**	137	Weighted average of orthodontic attendances (national reference costs)
**Eye specialist**	117	Paediatric ophthalmologist (national reference costs)
**Urologist**	133	Weighted average of urology attendances (national reference costs)

Table 3. **Unit costs for hospital stays and emergency services**

**Paediatric inpatient stay**		
Rotavirus complications	566	Weighted average of paediatric gastroenteritis elective inpatient attendances (national reference costs)
Minor injuries	1032	Weighted average of paediatric minor injury inpatient attendances (national reference costs)
Cardiology	1551	Weighted average of paediatric cardiology inpatient attendances (national reference costs)
**A&E**	218	Weighted average of A&E attendances (National reference costs)

Wider Societal Costs

Wider societal costs include out of pocket costs, the cost of education support, voluntary services and the cost of days off from school. We report wider service use and days off school by arm. When reporting days off from school we report total days off as well as the number of days off which participants attributed as being related to Tourette syndrome.

Out of pocket costs are calculated using the amount reported by parents in the CA-SUS. Wider service use is calculated using online sources shown in Table 4. The cost of days off from school are calculated using a human capital approach based on the cost of childcare for each day a participant has reported taking off school.

Table 4. **Unit costs for wider societal costs**

Item	Unit cost (£)	Source
**Education Support**		
Individual Tuition at home	31	Per hour average collected from Survey of 2000 tutors run by “thetutor.com”
Individual tuition in a special unit	21	Cost per hour based on average annual salary from National Careers Service (NCS), a 37-hour work week and 46·4 working weeks in a year.
Education welfare officer	18	Cost per hour based on average annual salary from NCS, a 37-hour work week and 46·4 working weeks in a year.
Classroom assistant	9	Cost per hour based on Office for National Statistics (ONS) Standard Occupational Classification (SOC) 2019 weekly wage and a 37-hour work week.
Support from Learning mentor	9	Cost per hour based on ONS SOC 2019 weekly wage and a 37-hour work week.
**Voluntary Services**		
Tourette’s Action	10	Unit cost for any average contact provided by Tourette’s Action
Day care centre	38	PSSRU 2019 cost per day
Early years support worker	10	Cost per hour based on average annual salary from NCS, a 37-hour work week and 46·4 working weeks in a year.
Local Self-help group	26	Assumed cost to be the same as parent support groups in the community.
**Days off school**	83	Daily wage of a childminder based on ONS SOC 2019.

Utilities

Utility tariffs are calculated from responses to the CHU-9D, [5] a child specific patient reported outcome measure validated for use in the calculation of Quality Adjusted Life Years (QALYs) and applying the algorithm developed by Stevens.[6]

The CHU-9D was completed as a patient reported outcome by the child and a proxy reported outcome asking about the child by a parent/carer at baseline, 3 and 6 months.

It is likely that the time-horizon of the trial data of 6 months is of insufficient duration to capture the full benefit of the intervention, whereas a more appropriate time horizon for a full economic evaluation would be a minimum of 18 months. As a result, we only report descriptive statistics for utilities to provide information of the potential suitability of the CHU-9D in a decision model using 18-month data and not for the calculation of QALYs.

Statistical Analysis

Analyses were pre-specified in a health economics analysis plan (HEAP).

We have reported complete case descriptive statistics for the percentage of participants that used each type of resource and mean number of contacts for the participants that used them. The mean difference in costs and utilities, 95% confidence interval for each resource use type was calculated using regression analysis adjusting for baseline costs, with site as a covariate and bias corrected bootstrapping with 1,000 iterations for complete cases (complete resource use at baseline, 3-and 6-month follow-up).

We assumed data was missing at random (MAR). Predictors of missingness were explored, with site identified as the only predictor of missingness. In line with the statistical analysis of the primary outcome we have conducted a complete case analysis adjusting for predictors of missingness.

Incremental cost-effectiveness ratio (ICER)

We explore the mean incremental cost per point reduction in YGTSS-TTSS between the ERP arm and psychoeducation arm from the health and social care cost perspective at 6 months. Costs include total healthcare resource use per participant and variable costs per participant for both trial arms. For the intervention arm, costs include the cost for the platform, therapist time, supervision and training. For the psychoeducation arm we do not include any platform costs, assuming that this information would be available as standard. The incremental cost per point reduction in YGTSS-TTSS was calculated using seemingly unrelated regression (SUR; Stata command SUREG) to account for any potential correlation between costs and outcomes.

Cost-effectiveness acceptability plane (CEP) and Cost-effectiveness acceptability curve (CEAC)

The adjusted, bootstrapped, SUR YGTSS-TTSS and costs data were used to calculate the probability that the ERP intervention is cost-effective compared to the psychoeducation for a range of cost-effectiveness threshold values. A cost-effectiveness plane of the bootstrapped results is also reported.

Sensitivity Analyses

Sensitivity analyses were conducted to explore the impact of different assumptions regarding the cost the intervention on the potential cost-effectiveness of the ERP intervention compared with the psychoeducation arm. Two assumptions were changed:

- Applying a licence fee per participant instead of a fixed cost per year based on a UK iCBT digital intervention.[7] We apply different licence fees starting at £25 to explore how this impacts the probability of cost-effectiveness.
- Assuming fortnightly supervision instead of weekly supervision of therapists delivering the intervention.

Results

Cost of BiP platform

Fixed cost:

***Table 5.* Fixed costs of BiP platform provided by Swedish team**

Item	Cost (£)
Cost per year in SEK	96000
Cost per year in GBP September 2020	8494
**Fixed cost of platform per participant GBP** (Number of participants using platform = 222)	**38**

Variable costs:

***Table 6.* Variable costs of running BiP platform**

	Intervention (ERP) (N=111)		Control (Psychoeducation) (N=111)	
	Mean (£)	S.D. (£)	Mean (£)	S.D. (£)
Child	3·36	1·85	2·54	1·46
Parent 1	3·46	1·95	3·07	1·52
Parent 2	0·04	0·33	0·01	0·10
Total	6·87	3·54	5·62	2·62

For variable login costs, the mean cost per patient was £1·25 higher (95% CI £0·46 to £2·04) for the ERP arm compared to the psychoeducation. The mean cost of the platform per participant in the intervention arm was therefore £39·25.

Cost of therapist support

Fixed cost:

***Table 1.* Fixed cost of therapist support**

Item	Description	Grade	Total Costs (£)
Independent training	Total of 10 hours of independent time reported	1 grade 4 RA<br>1 grade 5 Nurse	177
Therapist time to attend supervision	Weekly supervision lasting an hour over 108 weeks	1 grade 4 RA<br>3 grade 5 Nurses	7825
Delivery of supervision	Weekly supervision lasting an hour over the 108 weeks	1 grade 8c clinician<br>1 grade 7 Nurse practitioner	6894
Total cost of supervision and training			14896
**Fixed cost of therapist support per participant** (Number of participants using the platform = 222)			**66**

Variable cost:

Table 2: **variable cost of therapist support**

	Intervention (ERP) (N=111)		Control (Psychoeducation) (N=111)	
	Mean (£)	S.D. (£)	Mean (£)	S.D. (£)
Therapist time				
Child	18	9	16	9
Parent 1	25	13	22	10
Parent 2	0·29	2	0·09	1
Total	43	20	38	17
Phone cost				
Child	0·01	0·14	0	0·02
Parents	0·29	1	0·27	1
Parent and child	0·02	0·16	0·02	0·16
Total	0·33	1	0·29	1
**Total therapist cost**	**43**	**20**	**38**	**17**

For variable therapist support costs, the mean cost per patient was £5·03 higher (95% CI £0·88 to £9·97) for ERP compared with psychoeducation. The mean cost of therapist support for the intervention was £71·03.

In total, the mean cost of the intervention per participant was £110.

Total Health and social Care Resource use

Table 9 reports the mean number of contacts and SD for those who have used the service for specialist tic services.

Other service use and medication use are reported in tables 10 and 11. There was no significant difference in costs between the two group with a mean per patient difference in total resource use cost at 6 months of £63 (95% CI -£158 to £284) for the ERP arm compared with the psychoeducation (see Table 12).

***Table 3.* Use of specialist tic services**
***mean number of contacts of those with non-zero contacts**

		**Baseline**		**Total at 6 months**	
		Intervention (ERP) (N=112)	Control (Psychoeducation) (N=112)	Intervention (ERP) (N=93)	Control (Psychoeducation) (N=93)
Contacts with specialist tic clinic	N (%)	9 (8%)	5 (4%)	4 (4%)	8 (9%)
	Mean* (SD)	1 (0·2)	1 (0·11)	1 (1)	2 (2)
CAMHS	N (%)	34 (30%)	28 (25%)	25 (27%)	33 (35%)
	Mean* (SD)	3 (1)	2 (0)	5 (5)	3 (5)
Paediatrician in Hospital	N (%)	5 (4%)	15 (13%)	16 (17%)	15 (16%)
	Mean* (SD)	1 (0)	1 (0)	1 (1)	1 (0)
Paediatrician in Community setting	N (%)	2 (2%)	5 (4%)	5 (5%)	5 (5%)
	Mean* (SD)	1 (0)	1 (0)	2 (1)	1 (0)
Child and adolescent psychiatrist	N (%)	2 (2%)	4 (4%)	2 (2%)	8 (9%)
	Mean* (SD)	2 (1)	2 (1)	5 (4)	2 (2)
Neurologist	N (%)	2 (2%)	4 (4%)	6 (6%)	5 (5%)
	Mean* (SD)	1 (0)	1 (0)	3 (4)	1 (0)
Psychologist	N (%)	1 (1%)	2 (2%)	2 (2%)	4 (4%)
	Mean* (SD)	2 (N/A)	6 (6)	2 (1)	1 (1)
Speech and language therapist	N (%)	3 (3%)	2 (2%)	6 (6%)	1 (1%)
	Mean* (SD)	1 (1)	1 (0)	4 (5)	12 (·)
Occupational therapist	N (%)	2 (2%)	2 (2%)	5 (5%)	3 (3%)
	Mean* (SD)	1 (0)	3 (2)	6 (8)	1 (0)

***Table 4.* Other service use**
***mean number of contacts of those with non-zero contacts**

		Baseline		Total at 6 months	
		Intervention (ERP) (N=112)	Control (Psychoeducation) (N=112)	Intervention (ERP) (N=93)	Control (Psychoeducation) (N=93)
Contacts with professionals in the community	N (%)	75 (67%)	69 (62%)	65 (70%)	75 (81%)
	Mean* (SD)	4 (1)	6 (1)	5 (8)	4 (5)
Inpatient contacts	N (%)	3 (3%)	1 (1%)	1 (1%)	2 (2%)
	Mean* (SD)	1 (0)	1 (N/A)	5 (N/A)	1 (0)
A & E	N (%)	6 (5%)	11 (10%)	7 (8%)	9 (10%)
	Mean* (SD)	1 (0)	1 (0)	1 (1)	2 (1)

***Table 5.* Medication use**
(number and proportion of participants in each group taking medication)

		Baseline		Over 6 months	
		Intervention (ERP) (N=112)	Control (Psychoeducation) (N=112)	Intervention (ERP) (N=93)	Control (Psychoeducation) (N=93)
Clonidine	N (SD)	4 (21)	9 (31)	6 (25)	7 (25)
	%	4%	8%	6%	8%
Risperidone	N (SD)	0 (0)	3 (18)	0 (0)	3 (21)
	%	0%	3%	0%	3%
Aripiprazole	N (SD)	7 (27)	1 (11)	6 (23)	1 (10)
	%	6%	1%	6%	1%
**Any Tic Medication***	**N (SD)**	**14 (37)**	**16 (39)**	**17 (36)**	**17 (40)**
	**%**	**13%**	**14%**	**18%**	**18%**

*Above medications plus haloperidol, guanfacine & topiramate

***Table 6.* Healthcare Resource use cost in 2019/2020 GBP**

	**Baseline** Mean (SD) (£)		**Total at 6 months** Mean (SD) (£)		Difference at 6 months (95% CI) (£)
	Intervention (ERP) (N=112)	Control (Psychoeducation) (N=112)	Intervention (ERP) (N=93)	Control (Psychoeducation) (N=93)	
Specialist tic services	128 (248)	91 (168)	362 (615)	313 (567)	29·33 (-58·88 to 117·54)
Community Services	73 (166)	82 (178)	169 (397)	146 (237)	0·31 (-162·33 to 162·94)
Inpatient Services	10 (107)	14 (147)	31 (298)	15 (107)	19·37 (-38·96 to 77·71)
A & E	8 (41)	21 (77)	27 (97)	40 (128)	-6·05 (-37·61 to 25·51)
Medication Cost	13 (31)	13 (32)	33 (66)	27 (66)	7·68 (-6·52 to 21·89)
**Total Healthcare Resource Use**	**231 (361)**	**221 (333)**	**622 (1015)**	**541 (747)**	**63·29 (-157·79 to 284·37)**

Wider Societal Costs

Table 13 reports the proportion of participants in each arm that had contacts with education support services and voluntary services at baseline and over the following 6 months. The proportion of participants using these services was similar in both groups and there was no significant difference between the groups in terms of the cost of these services (Table 15).

***Table 7*. Education Support and voluntary services**
***mean number of contacts for those with non-zero contacts**

		**Baseline**		**Total at 6 months**	
		Intervention (ERP) (N=112)	Control (Psychoeducation) (N=112)	Intervention (ERP) (N=93)	Control (Psychoeducation) (N=93)
Education Support services	N (%)	47 (42%)	47 (42%)	46 (49%)	38 (41%)
	Mean* (SD)	47 (53)	34 (44)	47 (53)	63 (66)
Voluntary Services	N (%)	19 (17%)	18 (16%)	11 (12%)	9 (10%)
	Mean* (SD)	2 (1)	2 (3)	2 (2)	4 (8)

The number of days taken off school by the participants in both groups is shown in Table 14. Around 20% of participants reported taking days off that were related to Tourette syndrome symptoms or healthcare appointments due to the illness. Of those who reported taking days of due to the illness, there was no significant difference in the number of days taken off between groups. The cost of total days off school is reported in Table 15. There was no significant difference in costs.

***Table 8*: Days off from school**
***mean number of days off for those who took days off from school**

		Baseline		Total at 6 months		Difference at 6 months (95% CI)
		Intervention (ERP) (N=112)	Control (Psychoeducation) (N=112)	Intervention (ERP) (N=93)	Control (Psychoeducation) (N=93)	
Days off from School due to TS	N (%)	25 (22%)	20 (18%)	16 (17%)	19 (20%)	1·29 (-1·25 to 3·84)
	Mean* (SD)	6 (11)	8 (12)	11 (17)	9 (12)	
Days off from School total	N (%)	66 (59%)	52 (46%)	50 (54%)	52 (56%)	1·13 (-1·29 to 3·56)
	Mean* (SD)	5 (8)	5 (8)	8 (13)	6 (9)	

***Table 9.* Wider Costs**
***including out of pocket cost for specialist tic services, community services and education support**

	**Baseline** Mean (SD) (£)		**Total at 6 months** Mean (SD) (£)		Difference at 6 months (95% CI) (£)
	Intervention (ERP) (N=112)	Control (Psychoeducation) (N=112)	Intervention (ERP) (N=93)	Control (Psychoeducation) (N=93)	
Education Support	186 (515)	182 (573)	290 (657)	306 (725)	-9·30 (-163·90 to 145·30)
Voluntary Services	8 (29)	6 (22)	10 (40)	8 (31)	1·90 (-8·45 to 12·25)
Out of pocket costs*	6 (34)	112 (707)	192 (701)	277 (1077)	74·03 (-97·49 to 245·54)
Cost of days off school (total)	164 (340)	172 (497)	348 (854)	268 (583)	96·70 (-106·25 to 299·65)
**Total Wider Costs**	**365 (597)**	**472 (1161)**	**849 (1355)**	**859 (1394)**	**117·07 (-205·95 to 440·09)**
**Total Healthcare & Wider Costs**	**596 (793)**	**694 (1265)**	**1442 (2028)**	**1400 (1709)**	**203·16 (-241·03 to 647·34)**

Total wider costs were £117·07 higher on average for those in the intervention group (95% CI -£205·95 to £440·09). Once total healthcare costs were accounted for the difference was £203·16 (95% CI -£241·03 to £647·34). The difference in costs was not significant in either case.

Utilities

The follow up rate for the CHU-9D is lower than for the CA-SUS as this was collected using a different method to the primary outcome. At 6 months, for the child completed CHU-9D, utility scores were 0·046 (95% CI 0·013 to 0·080) higher for those in the ERP group compared to those in the psychoeducation group (Table 16). There was no statistically significant difference between arms for the parent completed CHU-9D (0·010 95% CI -0·023 to 0·043).

***Table 10*. Utilities using parent completed CHU-9D**

	Intervention (ERP)			Control (Psychoeducation)			Difference (95% CI)	P
	N	mean	S.D.	N	mean	S.D.		
Baseline	112	0·832	0·085	112	0·845	0·088		
3 months	89	0·853	0·091	92	0·822	0·107	0·033 (0·007 to 0·059)	0·010
6 months	64	0·849	0·106	76	0·841	0·110	0·010 (-0·023 to 0·043)	0·0072

***Table 11*. Utilities using child completed CHU-9D**

	Intervention (ERP)			Control (Psychoeducation)			Difference (95% CI)	P
	N	mean	S.D.	N	mean	S.D.		
Baseline	112	0·815	0·097	112	0·827	0·094		
3 months	86	0·866	0·102	89	0·831	0·117	0·038 (0·009 to 0·067)	0·012
6 months	63	0·853	0·118	66	0·814	0·122	0·046 (0·013 to 0·080)	0·056

ICER, CEP and CEAC

The incremental mean cost per patient of the ERP compared to the psychoeducation was £159 (95% CI -£53 to £370), with a mean of -2·65 points reduction on the YGTSS-TTSS (95% CI -4·55 to -0·74), with an incremental cost per reduction in YGTSS-TTSS (YGTSS-TTSS multiplied by -1) of £60 from a health and social care cost-perspective. There is a 99% probability that the ERP intervention is cost-effective compared with the psychoeducation for cost-effectiveness thresholds greater than £400, with a 79% probability that the ERP is cost-effective compared to psychoeducation at a £100 threshold.

From a wider cost perspective, the incremental cost per point reduction in YGTSS-TTSS was £48·58, with a 95% probability of being cost effective compared with the psychoeducation control at cost-effectiveness threshold of £400.

Figure 1: CEP
(Please note the ICER has not been multiplied by -1 so the interpretation of the plane is reversed)

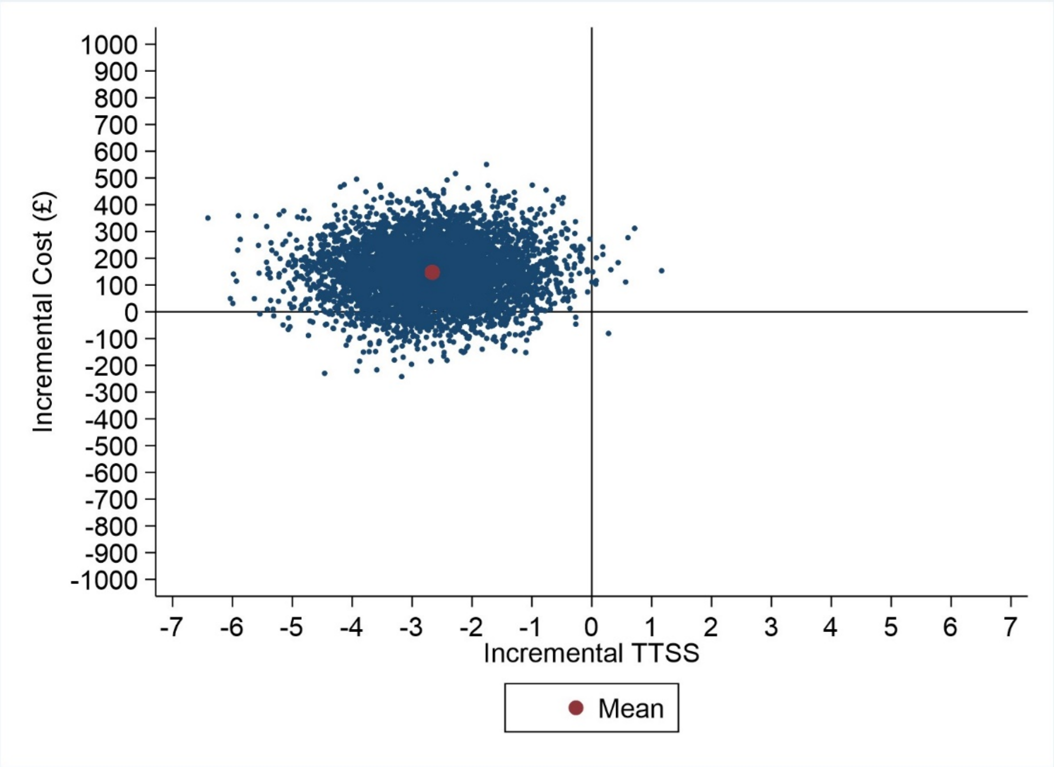

Figure 2: CEAC

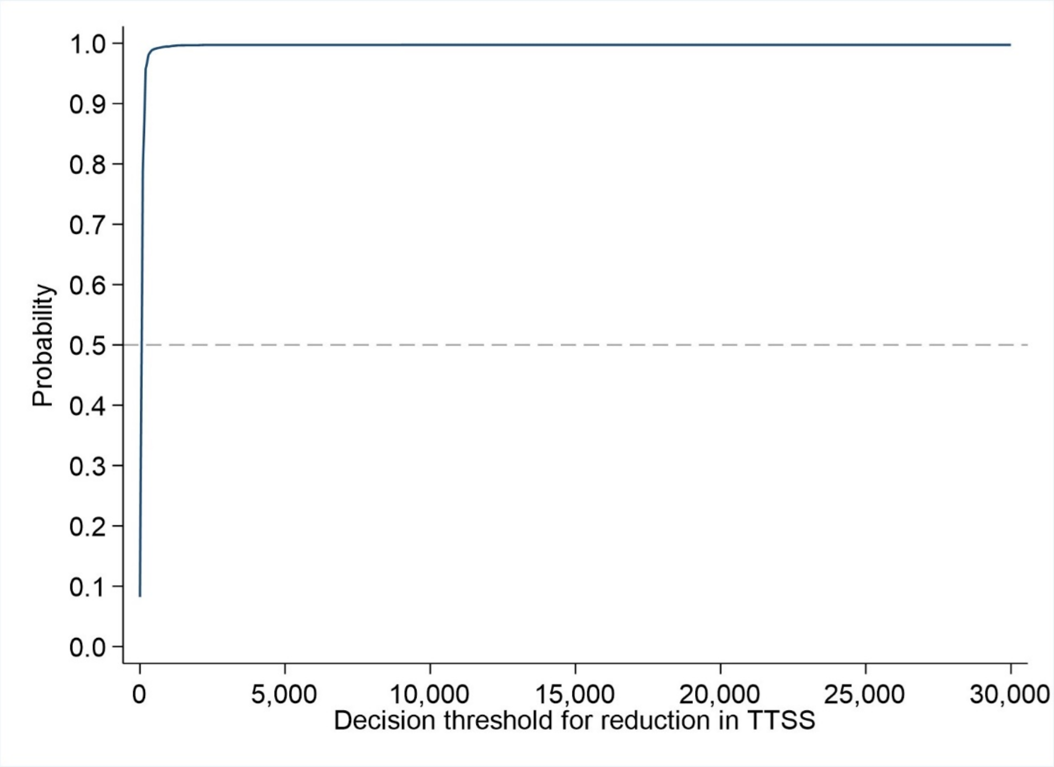

Sensitivity Analyses

In order to explore how different costing models for the platform impact the cost-effectiveness of the intervention we applied a licence fee starting at £25 per participant and increasing by £10 increments until £165. At a threshold value of £100 per point reduction in YGTSS-TTSS the probability of cost-effectiveness falls below 50% at a licence fee per participant of £145 or more. The probability that the ERP intervention is cost-effective compared to the psychoeducation at a cost-effectiveness threshold of £400 when applying the £145 licence fee is 97%.

Figure 3: Impact of different licence fees on probability of cost-effectiveness at a threshold of £100 per point reduction in YGTSS- TTSS

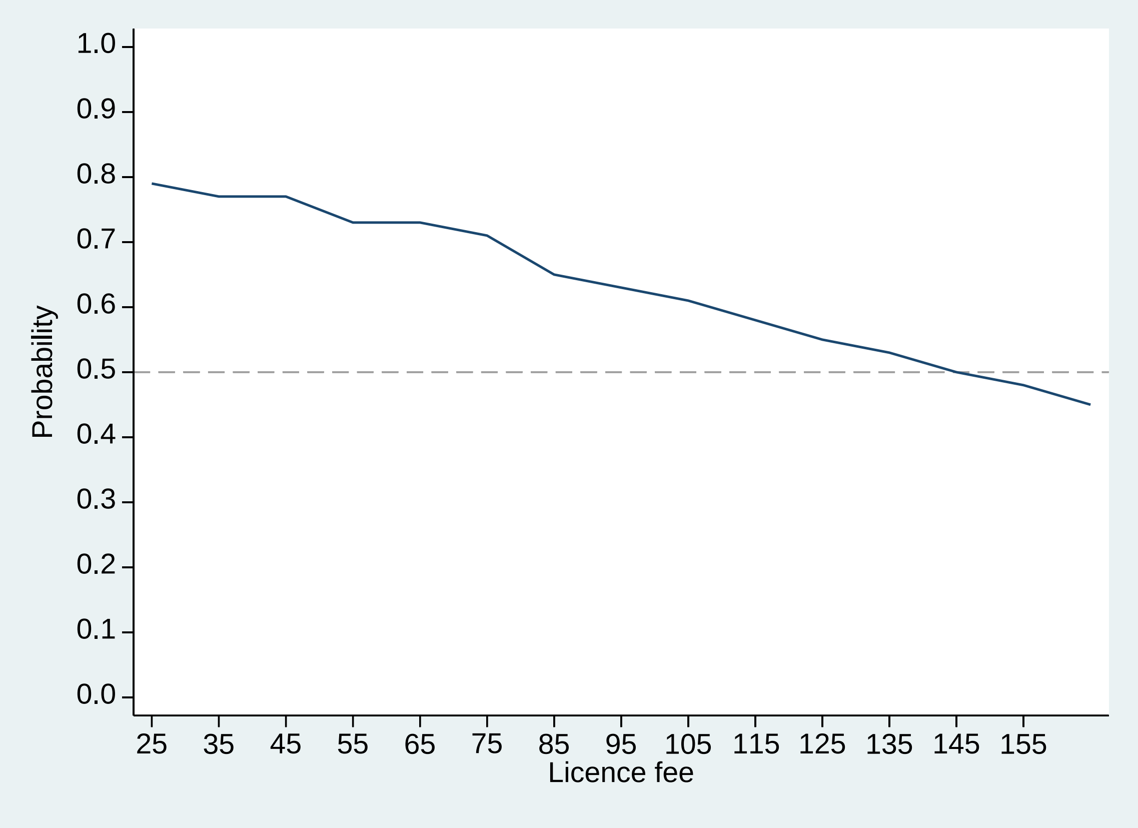

As therapists were trained during supervision, it is feasible to assume that once fully trained and familiar with the delivery of the intervention only fortnightly supervision would be necessary. When applying fortnightly supervision, the fixed cost of the intervention falls from £104 to £71. This results in an incremental cost-effectiveness ratio of £54 per point reduction in YGTSS-TTSS and probability of 82% of being cost-effective compared with the psychoeducation at a threshold of £100.

Discussion

Based on our exploratory economic evaluation, the therapist supported online remote behavioural intervention for tics cost £159 more on average per patient than online education, with the majority of the cost accounted for by the additional cost of the intervention. The mean additional cost per patient of the platform and therapist time including training and support was £110; this could be as low as £77 per participant if a move to fortnightly supervision is assumed. There was no evidence that the intervention had a significant impact on health and social care costs at 6 months compared to online education.

There was a statistically significant difference in favour of the intervention for the child completed CHU-9D, but not the proxy parent completed questionnaire, with the child completed version better reflecting the results for the primary outcome (YGTSS-TTSS). This mirrors research by Storch et al.[8] which compared tic severity to child and parent reported quality of life. They found that parent reported quality of life was weakly correlated to tic severity and that parent and child reported quality of life did not correlate as well in older children (>11 years).

This analysis is exploratory only to provide initial information on the potential cost of the ERP intervention compared to the psychoeducation and any anticipated impacts on costs and QALYs. The time horizon of the analysis is too short to conduct a full economic evaluation of the cost per QALY gained of the intervention compared to the psychoeducation given the 6-month follow-up period, with a maximum of 0·5 QALYs available. Instead, a full economic evaluation of the ERP intervention compared to the psychoeducation will be conducted using data from the 18-month trial follow-up. This will include a decision model projecting changes in YGTSS-TTSS into the future and mapping CHU-9D to the YGTSS-TTSS to calculate QALYs. It appears that this will be feasible and meaningful based on our initial results reported above given that the child completed CHU-9D and the YGTSS-TTSS seem to both be showing that the intervention results in better outcomes than online education. Our current finding that the mean incremental cost per point reduction in the YGTSS-TTSS is £60 is for illustrative purposes only given that it is not clear what the threshold for cost-effectiveness is for a point change in YGTSS-TTSS. There is a very high probability though that ERP is cost-effective compared to online education for very low thresholds of cost-effectiveness (>£100 per point reduction in YGTSS-TTSS). Further work will also need to be conducted on whether online education is the only comparator to the online behavioural intervention, as face-to-face therapy may also need to be included as a comparator in the decision model, with the view that it may be as effective, or potentially more effective than the online intervention, but likely for a significantly greater cost. The effectiveness and cost of face-to-face therapy will be obtained from the literature.

References

1. Byford S, Harrington R, Torgerson D, Kerfoot M, Dyer E, Harrington V, et al. Cost-effectiveness analysis of a home-based social work intervention for children and adolescents who have deliberately poisoned themselves: Results of a randomised controlled trial. *Br J Psychiatry* 1999;**174**(JAN.):56–62.

2. Harrington R, Peters S, Green J, Byford S, Woods J, McGowan R. Randomised comparison of the effectiveness and costs of community and hospital based mental health services for children with behavioural disorders. *Br Med J [Internet]* 2000 Oct 28 [cited 2020 Dec 16];**321**(7268):1047–50. Available from: http://www.bmj.com/

3. Barrett B, Byford S, Chitsabesan P, Kenning C. Mental health provision for young offenders: Service use and cost [Internet]. Vol. 188, *British Journal of Psychiatry Cambridge University Press* 2006 [cited 2020 Dec 16]; p. 541–6. Available from: https://www.cambridge.org/core.

4. Curtis L, Burns A. Unit Costs of Health and Social Care 2019; *Personal Social Sercvices Research Unit, University of Kent, Canterbury*.

5. Furber G, Segal L. The validity of the Child Health Utility instrument (CHU9D) as a routine outcome measure for use in child and adolescent mental health services. *Health Qual Life Outcomes* 2015; Feb 18;**13**(1).

6. Stevens K. Assessing the performance of a new generic measure of health-related quality of life for children and refining it for use in health state valuation. *Appl Health Econ Health Policy* 2011;**9**(3):157–69.

7. Richards D, Enrique A, Eilert N, Franklin M, Palacios J, Duffy D, et al. A pragmatic randomized waitlist-controlled effectiveness and cost-effectiveness trial of digital interventions for depression and anxiety. *npj Digit Med [Internet]*. 2020;**3**(1). Available from: http://dx.doi.org/10.1038/s41746-020-0293-8

8. Storch EA, Merlo LJ, Lack C, Milsom VA, Geffken GR, Goodman WK, et al. Quality of life in youth with Tourette's syndrome and chronic tic disorder. *J Clin Child Adolesc Psychol* 2007;**36**(2):217–27.

Effect of the ERP intervention mid-treatment

Table 1. **Effect of the behavioural therapy intervention mid treatment**

	**Control (Psychoeducation) (N = 112)** Mean (SD)	**Intervention (ERP) (N = 112)** Mean (SD)	**Estimated difference (95% CI)**
Parent Tic Questionnaire (PTQ)	45˙3 (24˙3)	42˙8 (27˙0)	-3˙58 (-8˙33 to 1˙16)
Mood and Feelings Questionnaire (MFQ)	14˙4 (10˙6)	12˙7 (12˙0)	-˙56 (-3˙54 to 0˙41)

Notes*: Statistics are mean (SD) unless otherwise specified. SD = standard deviation. CI = confidence interval. Statistical models adjusted for the baseline measure of the outcome in question and site.*

Unplanned analysis investigating effect of common comorbidities on the intervention

Table 1. **Effect of the behavioural therapy intervention on the primary outcome by common comorbidities**

	**Control (Psychoeducation) (N = 112)**		**Intervention (ERP) (N = 112)**		**3 months follow up**	
	**N**	**Mean (SD)**	**N**	**Mean (SD)**	**Estimated difference (95% CI)**	**Interaction p value**
**Anxiety disorder**						
No anxiety disorder	77	25˙9 (7˙3)	71	23˙2 (8˙2)	-1˙78 (-3˙61 to 0˙05)	0˙204
Anxiety disorder	23	29˙8 (6˙4)	30	25˙5 (8˙1)	-4˙10 (-7˙18 to -1˙02)	
**Attention deficit hyperactivity disorder (ADHD)**						
No ADHD	78	26˙4 (7˙5)	79	23˙3 (8˙3)	-2˙24 (-4˙03 to -0˙45)	0˙906
ADHD	22	28˙3 (6˙5)	22	25˙8 (7˙6)	-2˙47 (-5˙85 to 0˙92)	

Notes*: Statistics are mean (SD) unless otherwise specified. SD = standard deviation. CI = confidence interval. Statistical models adjusted for the baseline measure of the outcome in question and site. Anxiety disorders include separation anxiety, specific phobias, social phobia, panic disorder, agoraphobia and post traumatic stress disorder (PTSD). At 3 months follow up, there were 12 missing observations (11%) for the primary outcome in the behavioural therapy arm compared to 11 (10%) in the online education arm.*
